# Annotation of additional evolutionary conserved microRNAs in CHO cells from updated genomic data

**DOI:** 10.1002/bit.25539

**Published:** 2015-04-08

**Authors:** Andreas B. Diendorfer, Matthias Hackl, Gerald Klanert, Vaibhav Jadhav, Manuel Reithofer, Fabian Stiefel, Friedemann Hesse, Johannes Grillari, Nicole Borth

**Affiliations:** ^1^ Department of BiotechnologyBOKU University of Natural Resources and Life SciencesViennaAustria; ^2^ ACIB GmbHAustrian Centre of Industrial BiotechnologyGrazAustria; ^3^ Biberach University of Applied SciencesBiberachGermany

**Keywords:** microRNA, Chinese hamster ovary cell, next‐generation sequencing

## Abstract

MicroRNAs are small non‐coding RNAs that play a critical role in post‐transcriptional control of gene expression. Recent publications of genomic sequencing data from the Chinese Hamster (CGR) and Chinese hamster ovary (CHO) cells provide new tools for the discovery of novel miRNAs in this important production system. Version 20 of the miRNA registry miRBase contains 307 mature miRNAs and 200 precursor sequences for CGR/CHO. We searched for evolutionary conserved miRNAs from miRBase v20 in recently published genomic data, derived from Chinese hamster and CHO cells, to further extend the list of known miRNAs. With our approach we could identify several hundred miRNA sequences in the genome. For several of these, the expression in CHO cells could be verified from multiple next‐generation sequencing experiments. In addition, several hundred unexpressed miRNAs are awaiting further confirmation by testing for their transcription in different Chinese hamster tissues. Biotechnol. Bioeng. 2015;112: 1488–1493. © 2015 The Authors. Biotechnology and Bioengineering Published by Wiley Periodicals, Inc.

## Introduction

Chinese hamster ovary (CHO) cells are important mammalian hosts for the production of biopharmaceuticals. Over the last 25 years, the optimization of growth, product quality and titer was mainly driven by the modification of media, feeding strategies, and biotechnological processes. More recently, with the availability of genomic sequences, cell engineering strategies have emerged as an alternative route to improve cell line performance (Lim et al., 2010; Xiao et al., 2014), although deeper insight into genetic, transcriptional and translational regulation is required to obtain full control over cellular metabolism (Jadhav et al., 2013; Kildegaard et al., 2013).

MicroRNAs (miRNAs) are small (18–24 nucleotides long) non‐coding RNAs (ncRNA) that are transcribed as a primary transcript (pri‐miRNA) by RNA polymerase II. These transcripts are cleaved in the nucleus by the RNAase Drosha, to produce a pre‐miRNA with a characteristic stem‐loop secondary structure and a size of about 50–80 nt. For the final miRNA sequence, pre‐miRNAs are processed by the enzyme Dicer and incorporated into the miRNA‐induced silencing complex (miRISC) and bind to mRNA transcripts in their 3′‐UTR. Binding of the complex triggers either inhibition of translation or mRNA cleavage. By this process, miRNAs are capable of negatively regulating protein translation (Gregory et al., 2004) and therefore constitute an important layer in the post‐transcriptional control of gene expression (Hobert, 2008).

In 2011, the first systematic approaches to miRNA annotation in CHO cells were reported (Hackl et al., 2011; Johnson et al., 2011). Subsequent studies were focused on providing insights into the importance of miRNAs for molecular pathways relevant to cell culture engineering, such as growth (Jadhav et al., 2014), apoptosis (Druz et al., 2011), and protein secretion (Barron et al., 2011; Loh et al., 2014).

Recent publications of genomic sequencing data from CHO cells (Xu et al., 2011) as well as that of *Cricetulus griseus* (CGR) (Brinkrolf et al., 2013; Lewis et al., 2013) now have provided a basis for refining the annotation of: (i) known expressed cgr‐miRs, (ii) the identification of additional expressed miRNAs in CHO cells, and (iii) the discovery of miRNAs without current evidence for transcription in CHO cell lines.

For the annotation of novel microRNAs, (Ambros et al. 2003) specified five conditions, of which a reasonable combination has to be met to count as valid microRNA. In this study we based our criteria on the identification of a distinct ∼22 nt RNA transcript and the phylogenetic conservation of mature and pre‐miRNA sequences.

To expand the list of miRNAs available as possible engineering tools, we searched for evolutionary conserved miRNA sequences from other species in four genomic datasets, identified their genomic locations and hairpin sequences, and confirmed the expression of some of these in CHO, using next‐generation transcriptome sequencing results. This improvement and expansion of sequence and expression information of cgr‐miRs will be useful for further functional investigation of miRNAs, to gain a better understanding of post‐transcriptional regulation in cellular pathways, and to explore the potential function of silent CHO miRNAs, for which no expression evidence could be found yet.

**Figure 1:**
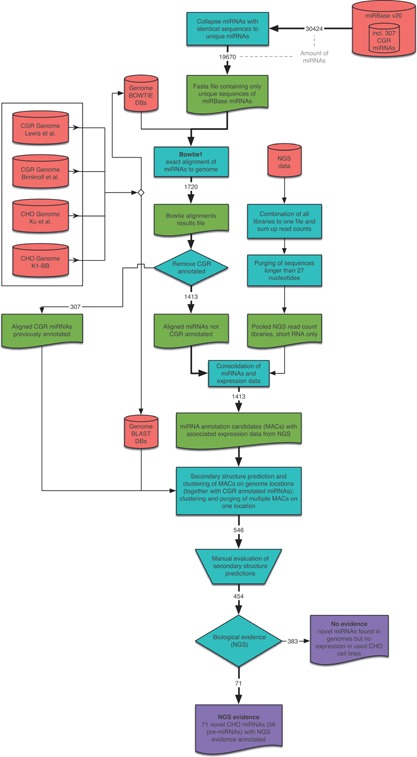
Bioinformatic pipeline flowchart. The main path of analysis is highlighted with a bold arrow from the top right to the bottom. Numbers on the arrows describe the amount of miRNAs processed in this step.

miRBase Version 20 (Griffiths‐Jones, 2004) contains 307 mature cgr‐miRs on 200 precursors. In order to identify and annotate novel cgr‐miRs, we based our search on evolutionary conservation of miRNAs by aligning all mature miRNAs to the available CGR/CHO genomic data, applying the workflow outlined in Figure 1.

Thereby, 1,720 unique mature sequences out of 19,670 miRBase v20 entries (all miRBase annotated miRNAs of any species) were aligned to at least one of the four genomes. The 307 already annotated cgr‐miRNAs were excluded, leaving 1,413 miRNA sequences as candidates for novel cgr‐miRNAs. Clustering of these sequences on genomic locations, to find similar, overlapping sequences that constitute only one possible new miRNA, reduced the number of sequences down to 546. These 546 candidates were then evaluated by prediction of their in silico secondary structure, whereby 454 sequences showed a pre‐miRNA like secondary stem loop structure. These putative novel cgr‐miRs are listed in Supplement 1.

For classification as novel cgr‐miRs, the next‐generation sequencing (NGS) read counts from new and existing datasets (Hackl et al., 2011) of these sequences were examined using more than five read counts as cut‐off for the existence of these miRNAs. Thereby, 383 sequences did not show expression under the constraints for valid NGS signals, leaving 71 NGS confirmed miRNAs.

Four of these 71 miRNAs were present on two genomic locations (75 possible novel miRNA locations) with different hairpin sequences (highlighted in blue in Supplement 1). A set of six pairs (miRNA‐5p/miRNA‐3p) could be matched on the same hairpin, (highlighted in green), giving 69 pre‐miRNA sequences. Thirteen of the new mature miRNA sequences were found on already annotated cgr‐pre‐miRNAs (see column “On hairpin with” in Supplement 1), thus complementing already annotated 5p or 3p miRNAs. Therefore, in summary, our study resulted in a total of 56 novel pre‐miRNA and 71 miRNA sequences that were added to the already annotated ones, extending the hamster miRNome to a total of 378 mature sequences (+23.1%), and 256 precursors (+28.0%).

The described process presents an easy and fast pipeline for the discovery of novel miRNAs from genomic data and next‐generation sequencing experiments. Recently published genomic data for *C. griseus* allowed the annotation of multiple conserved miRNAs from other species, by definition missing out on possible non‐conserved miRNAs present in the Chinese hamster.

**Figure 2:**
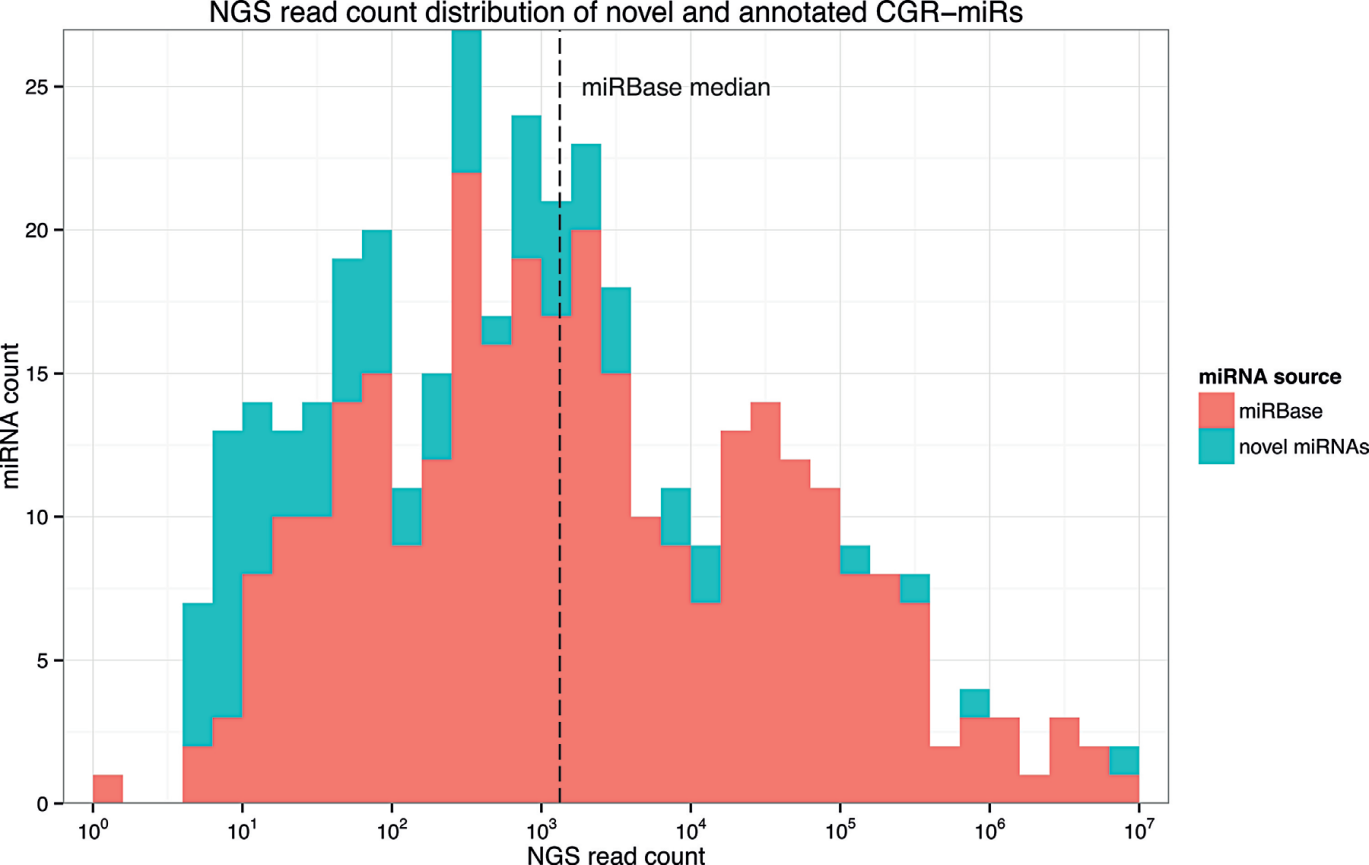
Read count distribution of miRBase annotated CGR and novel microRNAs showing a higher abundance of low read count microRNAs in the novel data set (77.8 % of novel miRNAs are below the miRBase median read count).

**Figure 3:**
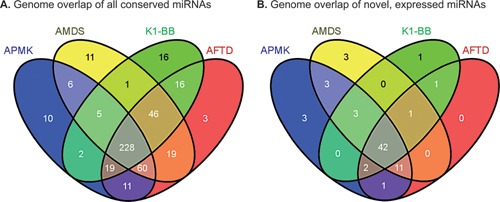
Venn diagrams showing the occurance of (**A**) all evolutionary conserved and (**B**) expressed, novel miRNAs in the four used genomic datasets.

In total, 71 novel expressed mature miRNAs and 56 pre‐miRNAs were added to the existing data. These novel miRNAs are mostly expressed at low levels, as their read counts were identified to be mainly below the median read count (77.8% below and 22.2% above) of already annotated cgr‐miRNAs (Fig. 2). In addition, we provide information on genomic loci of 345 mature miRNAs with no evidence for expression in CHO cells, but conserved homologous sequences in related species. These may be expressed in other tissue and cell types in the Chinese hamster, however , as confirmation of expression is required, they are currently not uploaded to miRBase. The occurrence of both the evolutionary conserved and expressed newly identified miRNAs in the four genomic datasets is shown in Figure 3. In both cases, the majority of miRNAs can be found in three or all genomic datasets.

A list of validated targets for these novel miRNAs (if available) is provided in Supplement 2. We grouped the pathways into cell engineering relevant categories. A list of pathways and miRNAs that possibly influence them is given in Table I. As these include process relevant cellular properties such as growth and apoptosis, the miRNAs may be relevant engineering targets.

**Table I:** Selected pathways possibly influenced by novel miRNAs. Data for miRNA protein interaction was derived from miRWalk (validated targets). DAVID was used to associate the proteins with pathways

Pathway category	miRNAs
Cell cycle related	mmu‐let‐7a‐1–3p, mmu‐miR‐106b‐5p, mmu‐miR‐126–5p, mmu‐miR‐127–3p, mmu‐miR‐202–5p, mmu‐miR‐211–5p, mmu‐miR‐216b‐5p, mmu‐miR‐24–1‐5p, mmu‐miR‐760–3p, hsa‐let‐7a‐3p, hsa‐let‐7c‐5p, hsa‐let‐7e‐3p, hsa‐let‐7e‐5p, hsa‐miR‐148a‐3p, hsa‐miR‐18b‐5p, hsa‐miR‐192–3p, hsa‐miR‐20b‐5p, hsa‐miR‐217, hsa‐miR‐26a‐1–3p, hsa‐miR‐26a‐2–3p, hsa‐miR‐449a, hsa‐miR‐582–5p
Apoptosis related	mmu‐let‐7a‐1–3p, mmu‐miR‐106b‐5p, mmu‐miR‐126–5p, mmu‐miR‐150–5p, mmu‐miR‐30c‐1–3p, hsa‐let‐7a‐3p, hsa‐let‐7c‐5p, hsa‐let‐7e‐3p, hsa‐let‐7e‐5p, hsa‐miR‐192–3p, hsa‐miR‐30c‐2–3p, hsa‐miR‐449a, hsa‐miR‐451a
Cancer related	mmu‐let‐7a‐1–3p, mmu‐miR‐101a‐5p, mmu‐miR‐106b‐5p, mmu‐miR‐126–5p, mmu‐miR‐127–3p, mmu‐miR‐150–5p, mmu‐miR‐193a‐5p, mmu‐miR‐193a‐3p, mmu‐miR‐211–5p, mmu‐miR‐216b‐5p, mmu‐miR‐24–1‐5p, mmu‐miR‐296–5p, mmu‐miR‐30c‐1–3p, mmu‐miR‐760–3p, mmu‐miR‐99b‐3p, mmu‐miR‐99b‐5p, hsa‐let‐7a‐3p, hsa‐let‐7c‐5p, hsa‐let‐7e‐3p, hsa‐let‐7e‐5p, hsa‐miR‐148a‐3p, hsa‐miR‐18b‐5p, hsa‐miR‐192–3p, hsa‐miR‐20b‐5p, hsa‐miR‐217, hsa‐miR‐26a‐1–3p, hsa‐miR‐26a‐2–3p, hsa‐miR‐301b‐3p, hsa‐miR‐30c‐2–3p, hsa‐miR‐449a, hsa‐miR‐451a, hsa‐miR‐582–5p
Focal adhesion related	mmu‐let‐7a‐1–3p, mmu‐miR‐101a‐5p, mmu‐miR‐126–5p, mmu‐miR‐150–5p, mmu‐miR‐30c‐1–3p, mmu‐miR‐99b‐3p, mmu‐miR‐99b‐5p, hsa‐let‐7a‐3p, hsa‐let‐7c‐5p, hsa‐let‐7e‐3p, hsa‐let‐7e‐5p, hsa‐miR‐148a‐3p, hsa‐miR‐192–3p, hsa‐miR‐20b‐5p, hsa‐miR‐217, hsa‐miR‐26a‐1–3p, hsa‐miR‐26a‐2–3p, hsa‐miR‐30c‐2–3p, hsa‐miR‐449a, hsa‐miR‐451a, hsa‐miR‐582–5p
Jak‐STAT related	mmu‐let‐7a‐1–3p, hsa‐let‐7a‐3p, hsa‐let‐7c‐5p, hsa‐let‐7e‐3p, hsa‐let‐7e‐5p, hsa‐miR‐192–3p, hsa‐miR‐26a‐1–3p, hsa‐miR‐451a align="center"
Cytoskeleton related	mmu‐let‐7a‐1–3p, mmu‐miR‐101a‐5p, mmu‐miR‐126–5p, mmu‐miR‐127–3p, mmu‐miR‐150–5p, mmu‐miR‐216b‐5p, mmu‐miR‐24–1‐5p, mmu‐miR‐296–5p, mmu‐miR‐30c‐1–3p, mmu‐miR‐99b‐3p, mmu‐miR‐99b‐5p, hsa‐let‐7a‐3p, hsa‐let‐7e‐3p, hsa‐miR‐192–3p align="center"
MAPK related	mmu‐let‐7a‐1–3p, mmu‐miR‐101a‐5p, mmu‐miR‐106b‐5p, mmu‐miR‐126–5p, mmu‐miR‐127–3p, mmu‐miR‐150–5p, mmu‐miR‐193a‐5p, mmu‐miR‐193a‐3p, mmu‐miR‐216b‐5p, mmu‐miR‐296–5p, mmu‐miR‐30c‐1–3p, mmu‐miR‐99b‐3p, mmu‐miR‐99b‐5p, hsa‐let‐7a‐3p, hsa‐let‐7c‐5p, hsa‐let‐7e‐3p, hsa‐let‐7e‐5p, hsa‐miR‐148a‐3p, hsa‐miR‐18b‐5p, hsa‐miR‐192–3p, hsa‐miR‐217, hsa‐miR‐26a‐1–3p, hsa‐miR‐26a‐2–3p, hsa‐miR‐451a align="center"

Taken together, the cgr‐miRNAs identified here will enhance the use of miRNAs as tools for CHO cell engineering. They also pave the way for using miRNAs not transcribed in CHO cells to restore or alter biotechnologically relevant cell line characteristics using the correct hamster primary miRNA sequences for engineering. The positive effects of using endogenous in contrast to chimeric miRNA sequences was shown by Klanert et al. (2014) and highlights the importance of good and comprehensive genomic data for CHO cell engineering.

## Material and Methods

### Datasets

For the annotation of novel miRNAs, the mature sequences of all miRNAs available in the miRNA database (miRBase version 20 ‐ http://www.mirbase.org; Griffiths‐Jones, 2004) were used. This constitutes a set of 30,424 mature miRNAs which was merged down to 19,670 unique sequences.

These miRNAs were aligned to four genome assemblies, sequenced from CHO cells and *C. griseus* (CGR). The genomes were downloaded from GenBank (http://www.ncbi.nlm.nih.gov/genbank/) and are listed with their accession IDs in Table II. In addition, the unpublished genome assembly “K1‐BB” was used as described by Hackl et al. (2012).

**Table II:** Genome references and statistics used for the identification of evolutionary conserved microRNAs

	Lewis et al. (2013)	Xu et al. (2011)	Brinkrolf et al. (2013)	K1‐BB
Genome size (Gbp)	2.3	2.3	2.1	2.98
Scaffolds	7,468	109,151	28,749	11,400,490
x Coverage	89.1	130.0	70.0	17.1
Accession ID	AMDS00000000.1	AFTD00000000.1	APMK00000000.1	—
Source	CGR	CHO	CGR	CHO

Next‐generation sequencing (NGS) data were obtained by Illumina sequencing as described by Hackl et al. (2011) and is available at the Sequence Read Archive SRA (www.ncbi.nlm.nih.gov/sra/), accession number SRA024456.1. Cell lines sequenced included CHO‐DUXB11 (ATCC CRL‐9096), CHO‐K1 (ECACC‐CCL‐61), and two derivative recombinant cell lines producing a monoclonal antibody (CHO‐K1) and an EpoFc fusion protein (DUXB11). Samples were taken both from cells grown in the presence of 5% FCS and after adaptation to protein free medium. To further extend the amount of cultivation conditions and cell lines we sequenced additional cell lines. CHO‐DG44 (passage 13) cells were grown in a 2 L bioreactor in batch culture. Total RNA was extracted with Trizol reagent (Invitrogen, Carlsbad, CA) according to the manufacturer's recommendations. The RNA quality was tested with an Agilent chip (Agilent Technologies, Santa Clara, California). Labelling of the samples followed the standard Illumina TrueSeq protocol for small RNA (12 barcoded samples on one lane). Sequencing of ready to load libraries was conducted by GATC, Germany on an Illumina HiSeq Analyser. The NGS data files of these experiments are available at Gene Expression Omnibus GEO (http://www.ncbi.nlm.nih.gov/geo/), accession number GSE59838 and SRA, accession number SRP044946.

### Bioinformatic Processing

Bioinformatic processing followed a pipeline of freely available tools and self‐written scripts. The scripts are available upon request from the corresponding author. The pipeline is outlined in Figure 1.

The collapsed mature miRNA sequences from miRBase were aligned against each of the four genome databases using Bowtie v1.0.0 (Langmead et al., 2009) to find exact matches. This produced a list of annotated miRNAs that were present in the genomic sequences (see Supplement 1). MicroRNAs already annotated for CGR were filtered and stored in a separate file. For all other miRNAs that could be aligned to the genomes, the read counts from next‐generation sequencing experiments were consolidated into one file. Next‐generation sequencing read counts were summed up from different experiments to consider the different culture conditions. A read count of five reads was set as cutoff.

For these miRNA annotation candidates, the up and downstream sequences were extracted in the same length as in the originating species (assuming that not only the mature sequence is conserved, but also the precursor). For this step, a BLAST (Altschul et al., 1990) database was generated for each genome, allowing the fast retrieval of sequences from given positions in the genomes. A secondary structure prediction was performed with RNAfold v2.1.3 from the ViennaRNA package (Lorenz et al., 2011), giving a graphical representation of each folding in addition to its free energy. For each annotation candidate a results file (HTML) was generated, consolidating the secondary structure images and other information for quick evaluation in the next steps.

For most of the identified locations, more than one miRNA sequence from different species were found by clustering all aligned miRNA sequences on distinct locations in each of the four genomes. To ensure that these evolutionary conserved sequences were only considered once, these not completely identical sequences (they differ in length and/or are shifted some nucleotides in their position) were sorted for their NGS read count and the highest was kept as annotation candidate. The secondary structures were manually evaluated for common miRNA characteristics (low minimum free energy, stem‐loop motif, miRNA sequence located in the stem, and no large internal loops or bulges) and are shown in Supplement 3.

To investigate possible uses of the novel miRNAs, we looked at validated miRNA‐targets in the originating species (*H. sapiens* or *M. musculus*) with miRWalk (Dweep et al., 2011). The list of target proteins were clustered using the functional annotation clustering tool of DAVID (Huang et al., 2009a; Huang et al., 2009b) and the KEGG pathway database and then grouped into seven categories (Table II).

GK and VJ are supported by the FWF doctorate program “BioTop”, W1224. GK is supported by the Austrian Center of Industrial Biotechnology, a public‐private competence center funded by the Austrian FFG.

## Supporting information

Additional supporting information may be found in the online version of this article at the publisher's web‐site.

Supplement 1.

Supplement 2.

Supplement 3.
