# Supplementary material for: Annotation of additional evolutionary conserved microRNAs in CHO cells from updated genomic data
Source: Biotechnol Bioeng. 2015 Apr 8;112(7):1488–93. doi: 10.1002/bit.25539 (PMC4949662; doi:10.1002/bit.25539)

bta-miR-200a

Genome:

APMK

Location:

eedd5|gi|529520636|gb|
APMK01309605.1|:
14968-15053_Cricetulus_gr
iseus_strain_17A/
GY_chromosome_2_chr2_cont
ig_37210_whole_genome_sho
tgun_sequence

Mature sequence:

UAACACUGUCUGGUAACGAUGUU

Precursor sequence:

gggccucugugggcaucuuaccgga
cagugcuggauuucuugacuugacu
cUAACACUGUCUGGUAACGAUGUUc
aaaggugaccc

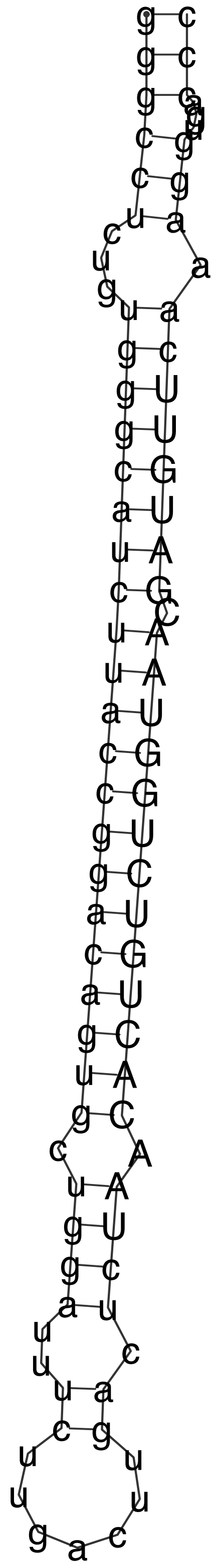

hsa-let-7a-2-3p

Genome:

APMK

Location:

f2795|gi|529542820|gb|APMK01299544.1|:c17267-17196_Cricetulus_griseus_strain_17A/GY_chromosome_4_chr4_contig_4632_whole_genome_shotgun_sequence

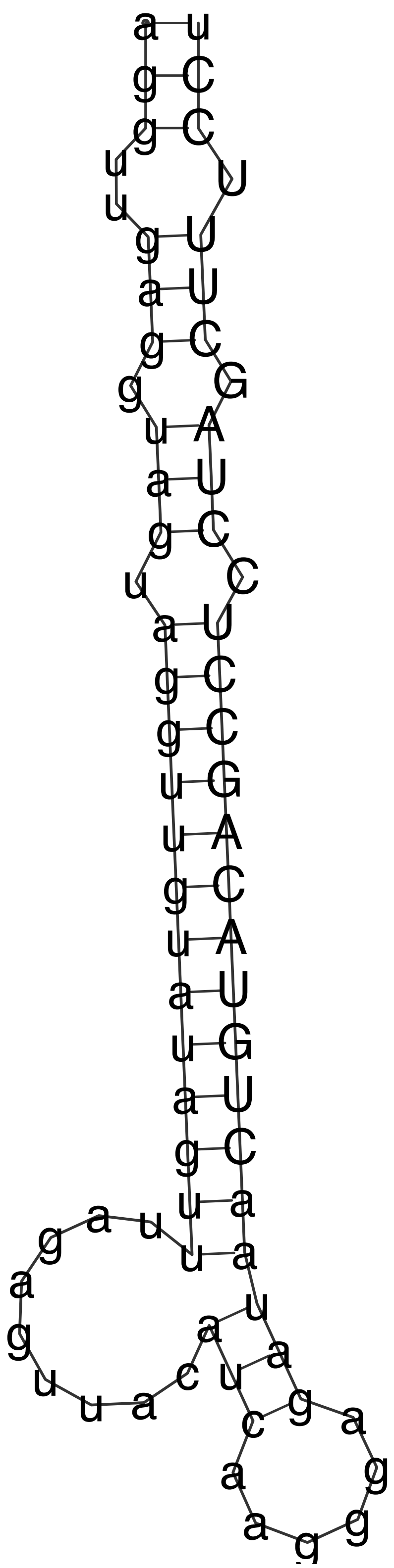

Mature sequence:

CUGUACAGCCUCCUAGCUUUCC

Precursor sequence:

agguugagguaguagguuguauaguuuagaguuacaucaagggagauaaCUGUACAGCCUCCUAGCUUUCCu

hsa-let-7a-3p

3a

Genome:

APMK

Location:

d0c12|gi|530049787|gb|
APMK01060022.1|:c20009-19
936_Cricetulus_griseus_st
rain_17A/
GY_chromosome_2_chr2_cont
ig_1047_whole_genome_shot
gun_sequence

Mature sequence:

CUAUACAAUCUACUGUCUUUC

Precursor sequence:

gggugagguaguagguuguaugguu
uugggcucugccccgcucugcggua
aCUAUACAAUCUACUGUCUUUCcu

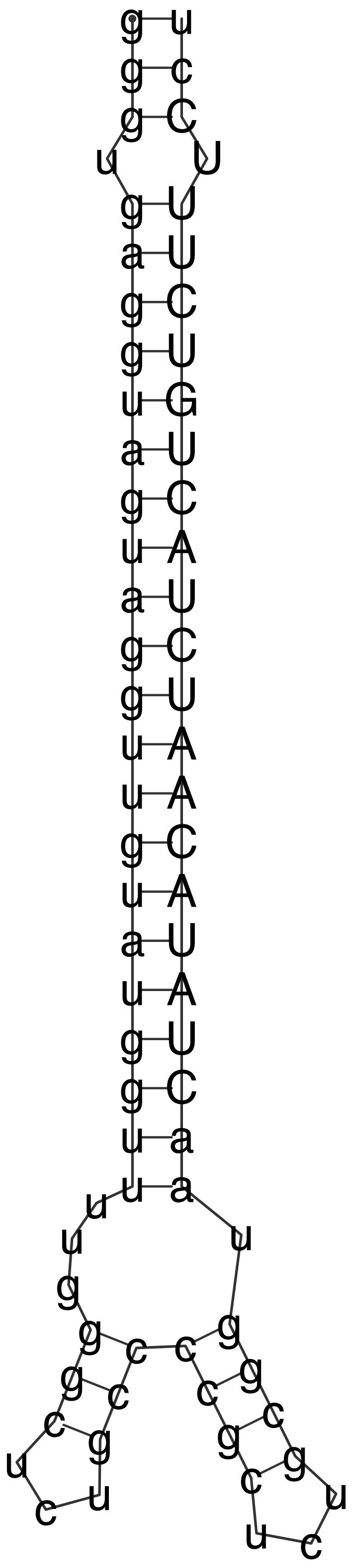

hsa-let-7a-3p

3b

Genome:

APMK

Location:

c4861|gi|529834860|gb|APMK01162609.1|:c5502-5429_Cricetulus_griseus_strain_17A/GY_chromosome_3_chr3_contig_8789_whole_genome_shotgun_sequence

Mature sequence:

CUAUACAAUCUACUGUCUUUC

Precursor sequence:

ugagguaguagguuguauaguuuuagggucacacccaccacugggagauaaCUAUACAAUCUACUGUCUUUCcu

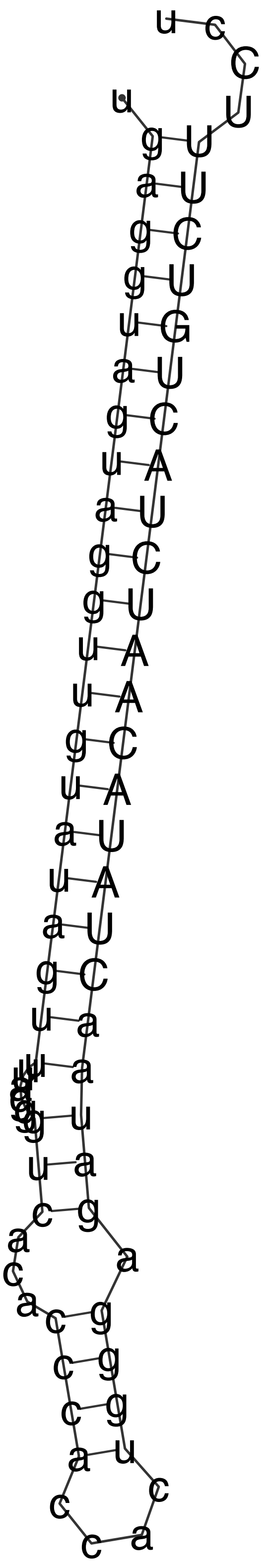

hsa-let-7c-5p

Genome:

APMK

Location:

eeccc|gi|529650272|gb|APMK01250788.1|:c1430-1347_Cricetulus_griseus_strain_17A/GY_chromosome_4_chr4_contig_19780_whole_genome_shotgun_sequence

Mature sequence:

UGAGGUAGUAGGUUGUAUGGUU

Precursor sequence:

ggguUGAGGUAGUAGGUUGUAUGGUUuagaguuacacccugggaguuaacuguacaaccuucuagcuuuccuuggagcac

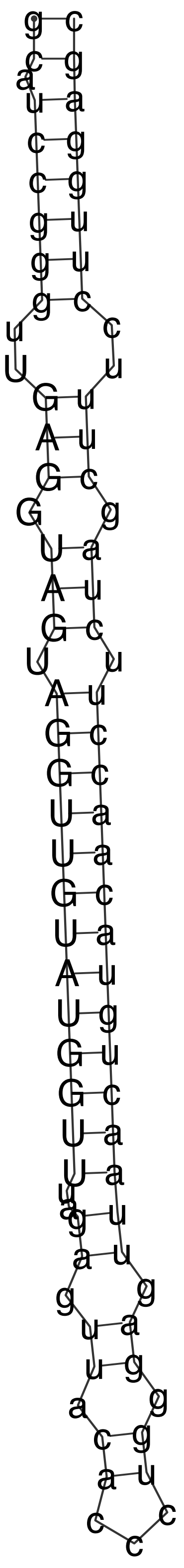

hsa-let-7c-5p

4b

Genome:

APMK

Location:

f67c8|gi|530049787|gb|APMK01060022.1|:c20016-19933_Cricetulus_griseus_strain_17A/GY_chromosome_2_chr2_contig_1047_whole_genome_shotgun_sequence

Mature sequence:

UGAGGUAGUAGGUUGUAUGGUU

Precursor sequence:

gccuuuggggUGAGGUAGUAGGUUGUAUGGUUuugggcucugccccgcucugcgguaacuauacaaucuacugucuuuccugaa

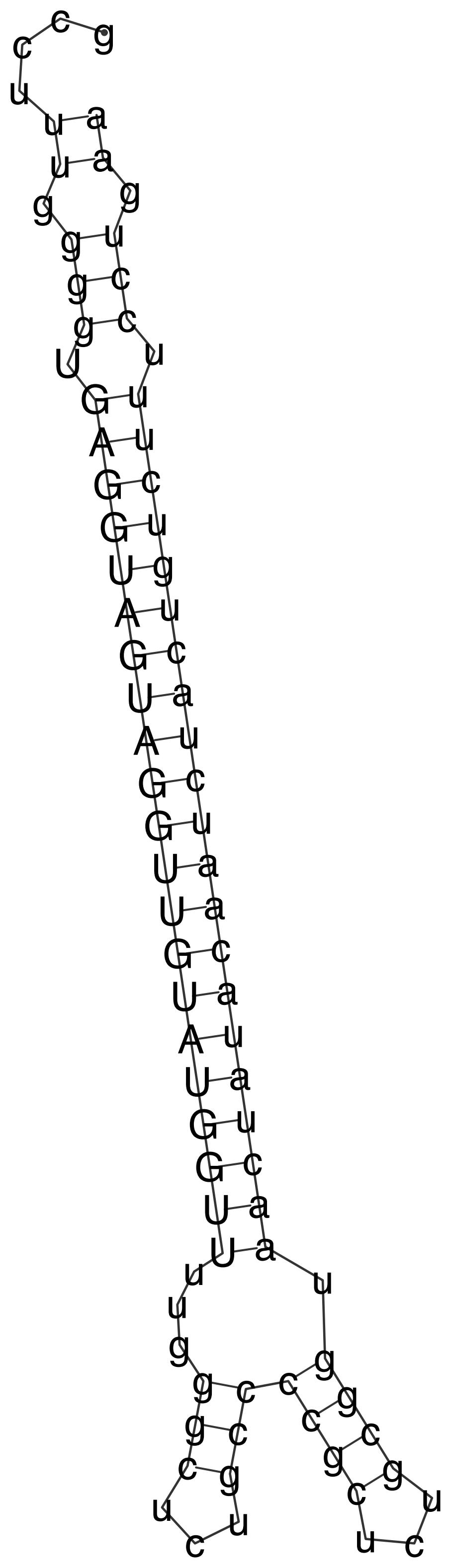

hsa-let-7e-3p

Genome:

APMK

Location:

cfcf3|gi|530137215|gb|APMK01015949.1|:1345-1423_Cricetulus_griseus_strain_17A/GY_unplaced_contig_5750_whole_genome_shotgun_sequence

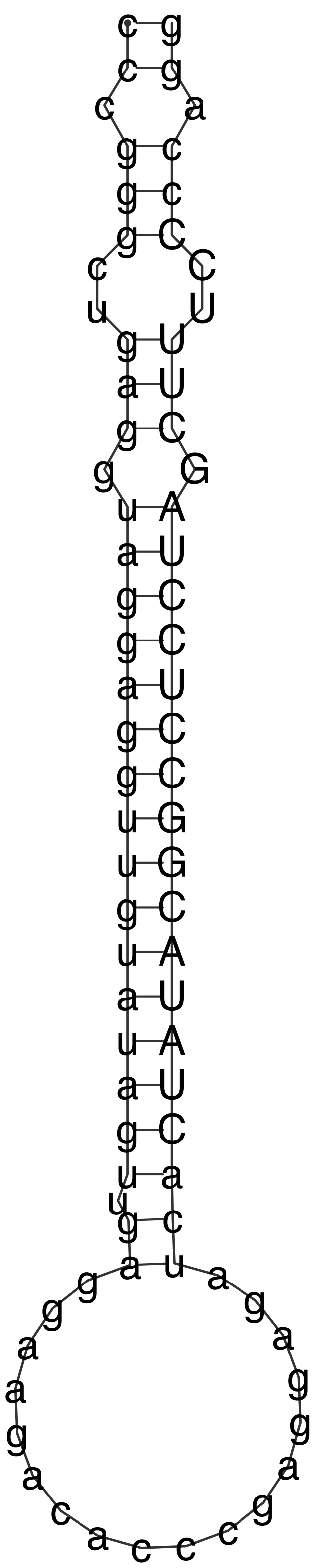

Mature sequence:

CUAUACGGCCUCCUAGCUUUCC

Precursor sequence:

cccgggcugagguaggagguuguauaguugaggaagacacccgaggagaucaCUAUACGGCCUCCUAGCUUUCCcagg

hsa-let-7e-5p

Genome:

APMK

Location:

b65a9|gi|530137215|gb|
APMK01015949.1|:
1345-1423_Cricetulus_gris
eus_strain_17A/
GY_unplaced_contig_5750_w
hole_genome_shotgun_seque
nce

Mature sequence:

UGAGGUAGGAGGUUGUAUAGUU

Precursor sequence:

cccgggcUGAGGUAGGAGGUUGUAU
AGUUgaggaagacacccgaggagau
cacuauacggccuccuagcuuuccc
cagg

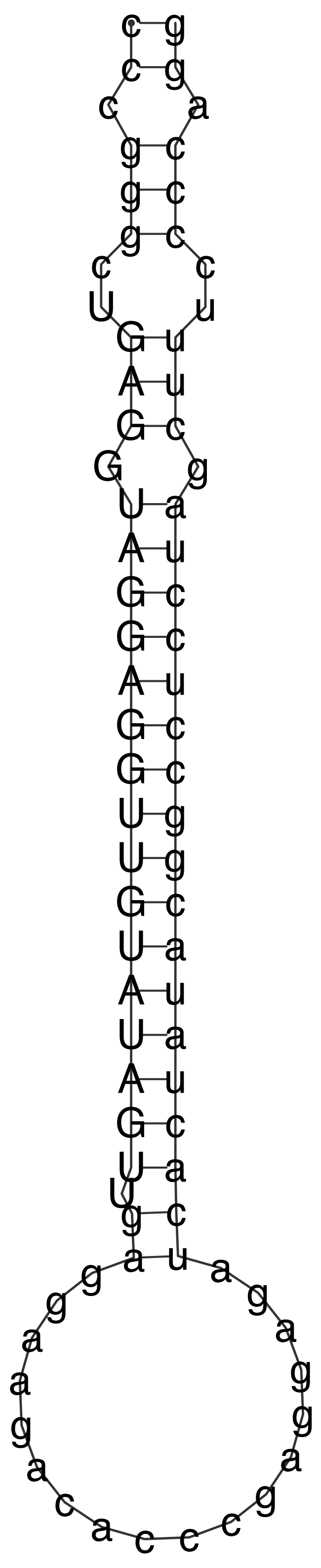

hsa-miR-148a-3p

Genome:

APMK

Location:

14d54|gi|529954026|gb|
APMK01103077.1|:
893-960_Cricetulus_griseu
s_strain_17A/
GY_chromosome_8_chr8_cont
ig_3157_whole_genome_shot
gun_sequence

Mature sequence:

UCAGUGCACUACAGAACUUUGU

Precursor sequence:

gaggcaaaguucugagacacuucga
cucugaguaugauagaagUCAGUGC
ACUACAGAACUUUGUcuc

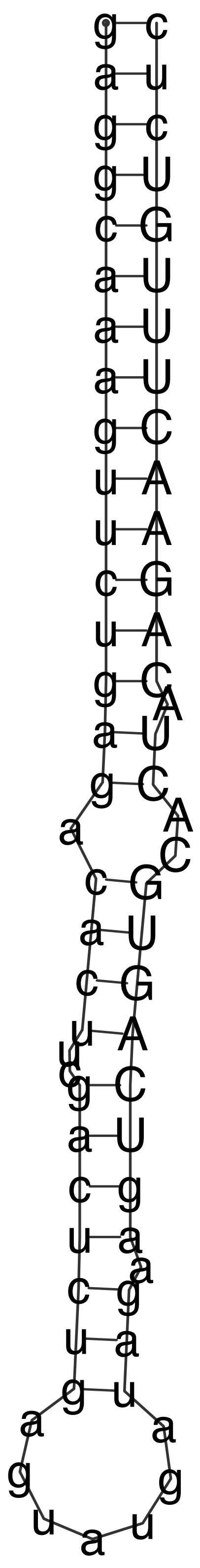

hsa-miR-192-3p

Genome:

APMK

Location:

010e9|gi|529816791|gb|
APMK01171231.1|:
10091-10200_Cricetulus_gr
iseus_strain_17A/
GY_chromosome_3_chr3_cont
ig_11718_whole_genome_sho
tgun_sequence

Mature sequence:

CUGCCAAUUCCAUAGGUCACAG

Precursor sequence:

gccaagauggagugcacagggcucu
gaccuaugaauugacagccagugcu
cuggucucuccucuggCUGCCAAUU
CCAUAGGUCACAGguauguucgccu
caaugccagc

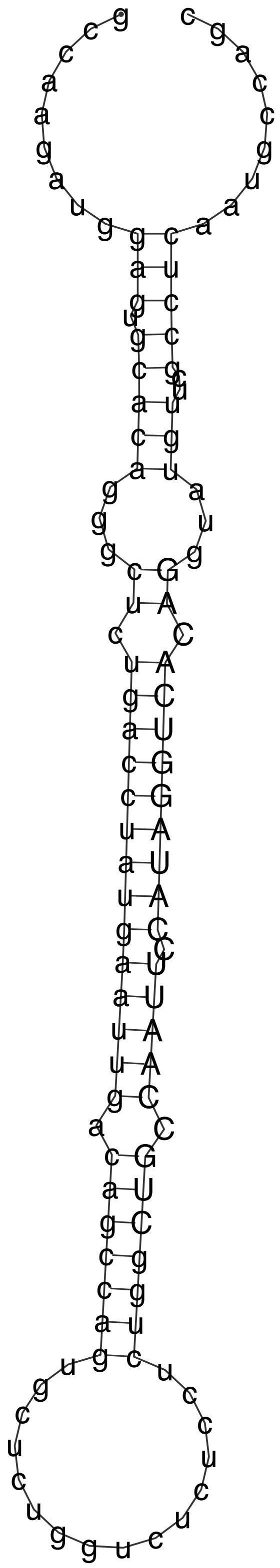

Genome:

APMK

Location:

efb8f|gi|529816791|gb|
APMK01171231.1|:
9906-9990_Cricetulus_gris
eus_strain_17A/
GY_chromosome_3_chr3_cont
ig_11718_whole_genome_sho
tgun_sequence

Mature sequence:

CCAGUGGGGCUGCUGUUAUCUG

Precursor sequence:

uggcugccacccccuguaacagcaa
cuccauguggaagugcccacuaguu
CCAGUGGGGCUGCUGUUAUCUGggg
uggaggcugg

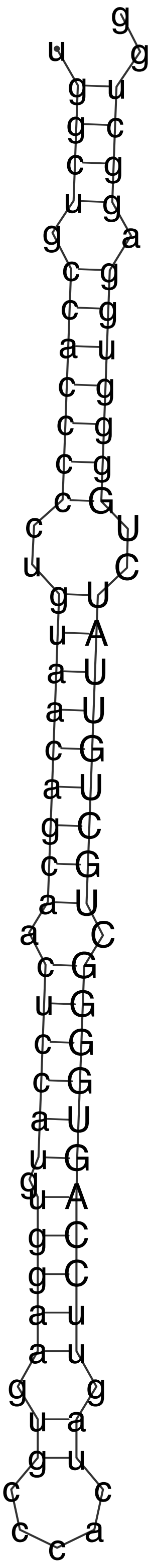

Genome:

APMK

Location:

61aeb|gi|529701335|gb|APMK01227117.1|:1368-1477_Cricetulus_griseus_strain_17A/GY_chromosome_1_chr1_contig_50998_whole_genome_shotgun_sequence

Mature sequence:

UACUGCAUCAGGAACUGAUUGGA

Precursor sequence:

ucaacacaguugucacaguuuuugaugucguagaUACUGCAUCAGGAACUGAUUGGAuaagacaaggucaccaucagucccuaaugcauuaccuucagcaucuaaaaaac

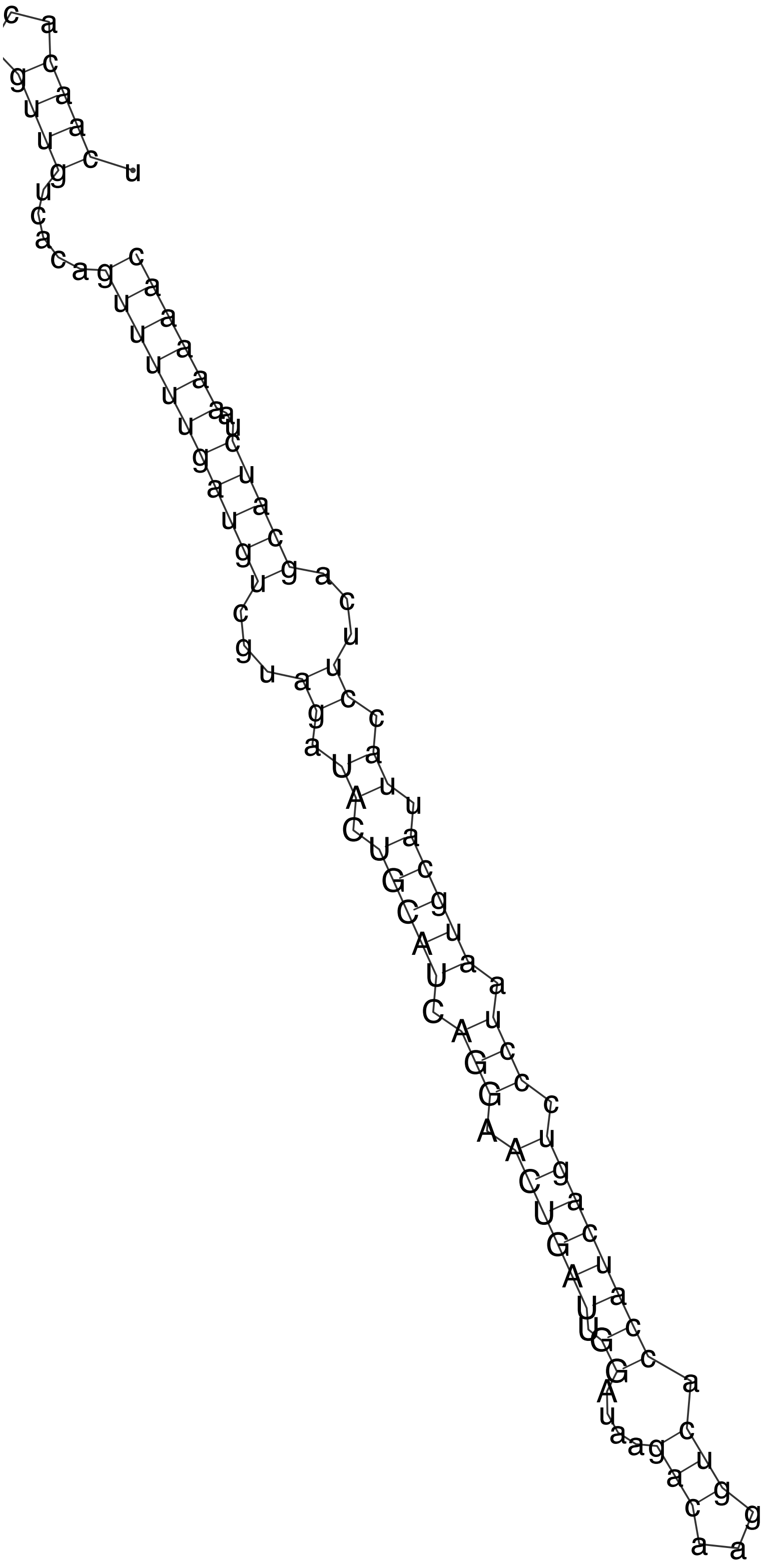

hsa-miR-219b-3p

Genome:

APMK

Location:

3e9d5|gi|530069208|gb|
APMK01050271.1|:c15298-15
211_Cricetulus_griseus_st
rain_17A/
GY_chromosome_6_chr6_cont
ig_2645_whole_genome_shot
gun_sequence

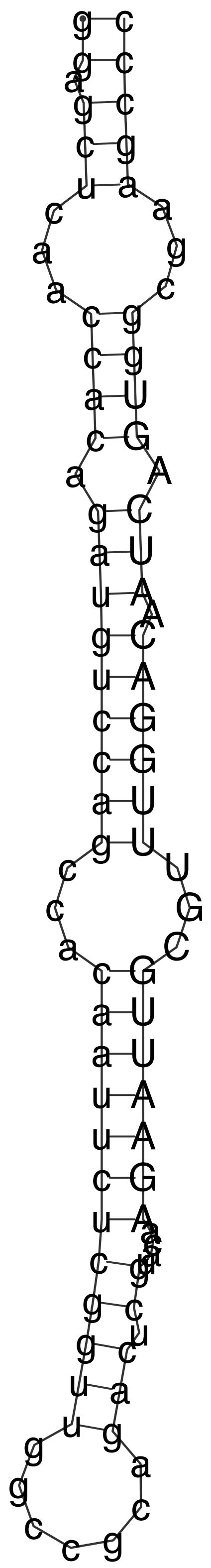

Mature sequence:

AGAAUUGCGUUUGGACAAUCAGU

Precursor sequence:

ggagcucaaccacagauguccagcc
acaauucucgguuggccgcagacuc
guacaAGAAUUGCGUUUGGACAAUC
AGUggcgaagccc

Genome:

APMK

Location:

6f43d|gi|529930098|gb|
APMK01114626.1|:c73-1_Cri
cetulus_griseus_strain_17
A/
GY_chromosome_4_chr4_cont
ig_17401_whole_genome_sho
tgun_sequence

Mature sequence:

CCUAUUCUUGGUUACUUGCACG

Precursor sequence:

guggccucguucaaguaauccagga
uaggcugugcaggucccaaggggCC
UAUUCUUGGUUACUUGCACGggg

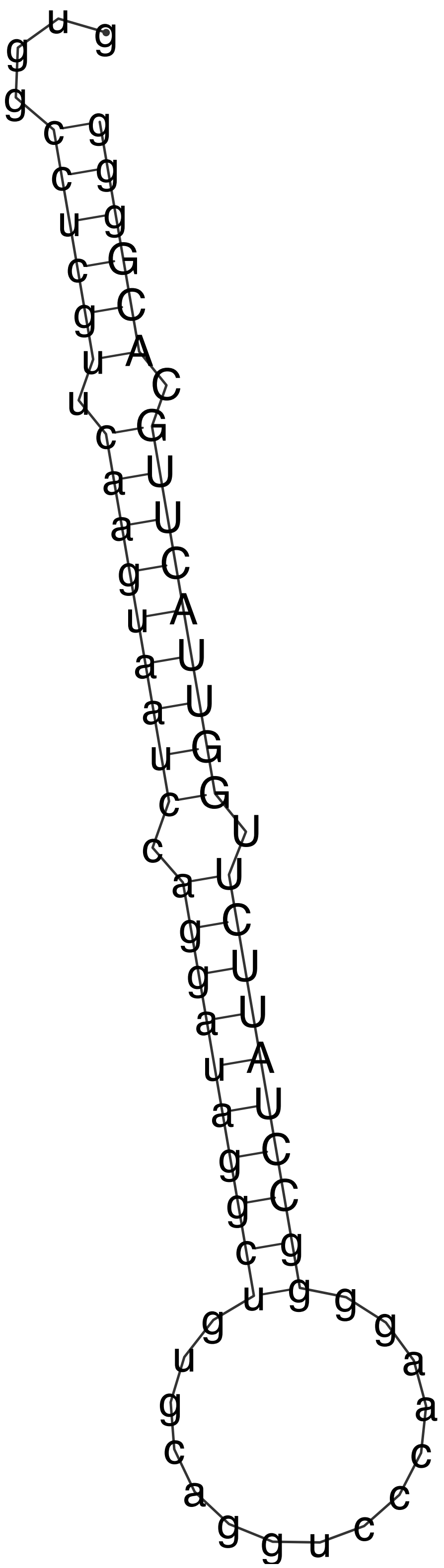

Genome:

APMK

Location:

0b552|gi|529852689|gb|
APMK01153754.1|:
3612-3695_Cricetulus_gris
eus_strain_17A/
GY_chromosome_1_chr1_cont
ig_49751_whole_genome_sho
tgun_sequence

Mature sequence:

CCUAUUCUUGAUUACUUGUUUC

Precursor sequence:

ggcuguggcuggauucaaguaaucc
aggauaggcuguuuccaugugugag
gCCUAUUCUUGAUUACUUGUUUCug
gaggcagcu

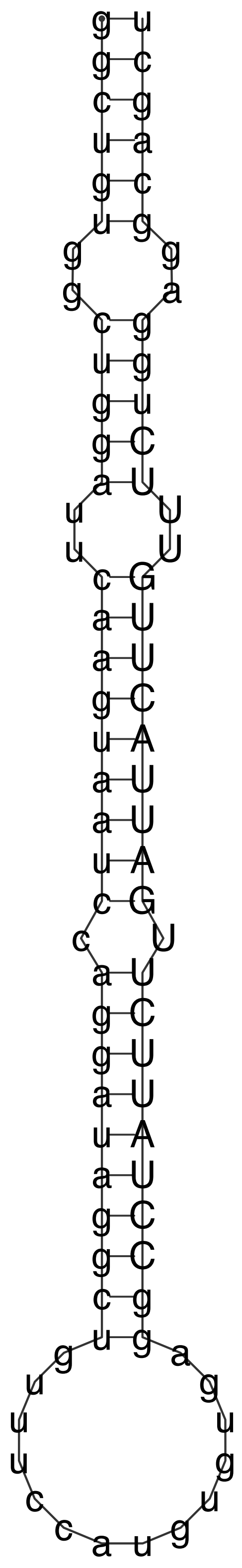

hsa-miR-301b

Genome:

```
APMK
```

Location:

```
033e7|gi|529926527|gb|
APMK01116334.1|:
443-520_Cricetulus_griseu
s_strain_17A/
GY_chromosome_4_chr4_cont
ig_21825_whole_genome_sho
tgun_sequence
```

Mature sequence:

```
CAGUGCAAUGAUAUUGUCAAAGC
```

Precursor sequence:

```
gcugcaggugcucugacuagguugc
acuacugugcucugagaagCAGUGC
AAUGAUAUUGUCAAAGCaucuggga
cca
```

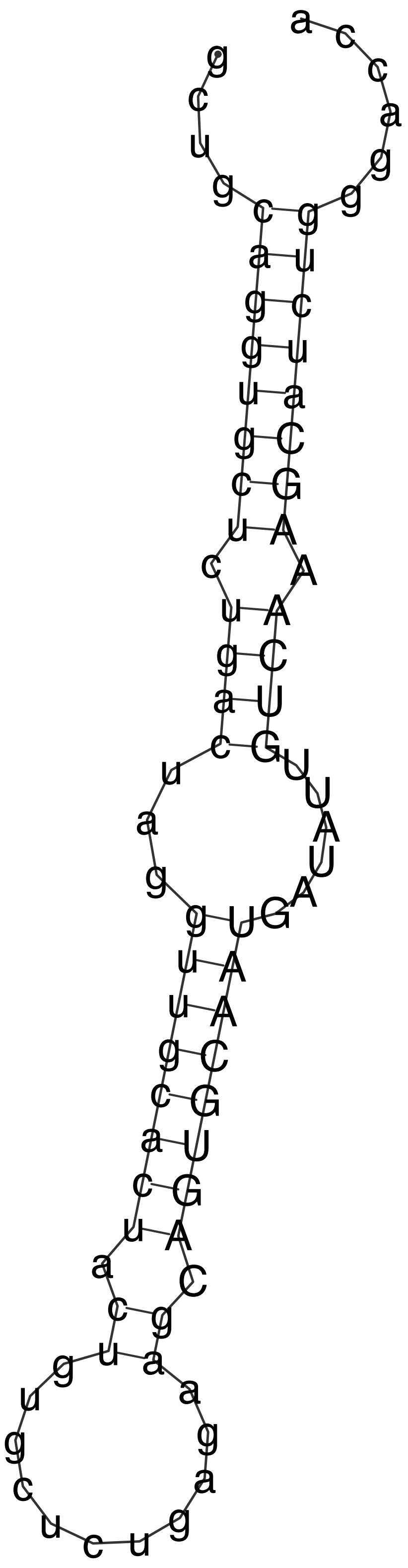

hsa-miR-30c-2-3p

Genome:

APMK

Location:

2190e|gi|529974421|gb|
APMK01093948.1|:
2503-2574_Cricetulus_gris
eus_strain_17A/
GY_chromosome_1_chr1_cont
ig_60553_whole_genome_sho
tgun_sequence

Mature sequence:

CUGGGAGAAGGCUGUUUACUCU

Precursor sequence:

agauacuguaaacauccuacacucu
cagcugugaaaaguaagaaagCUGG
GAGAAGGCUGUUUACUCUcucu

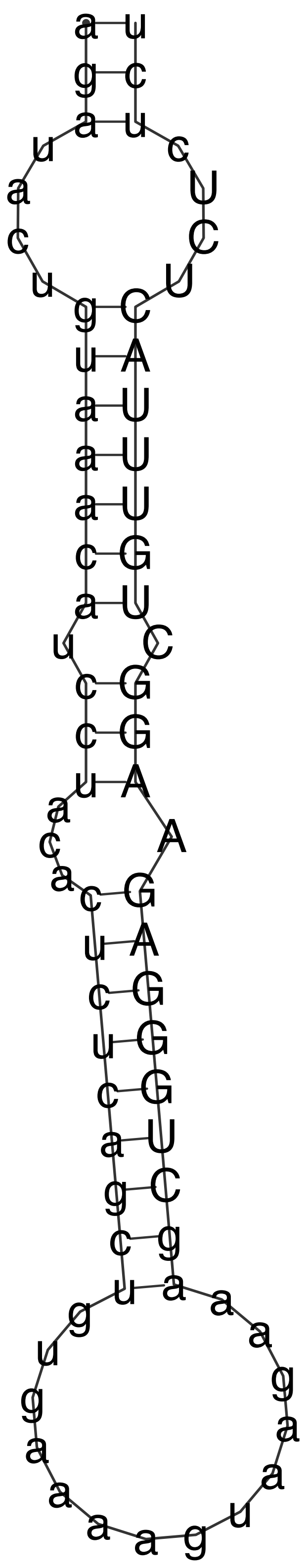

hsa-miR-3154

Genome:

APMK

Location:

c618d|gi|529673050|gb|APMK01239581.1|:c1013-930_Cricetulus_griseus_strain_17A/GY_chromosome_6_chr6_contig_2349_whole_genome_shotgun_sequence

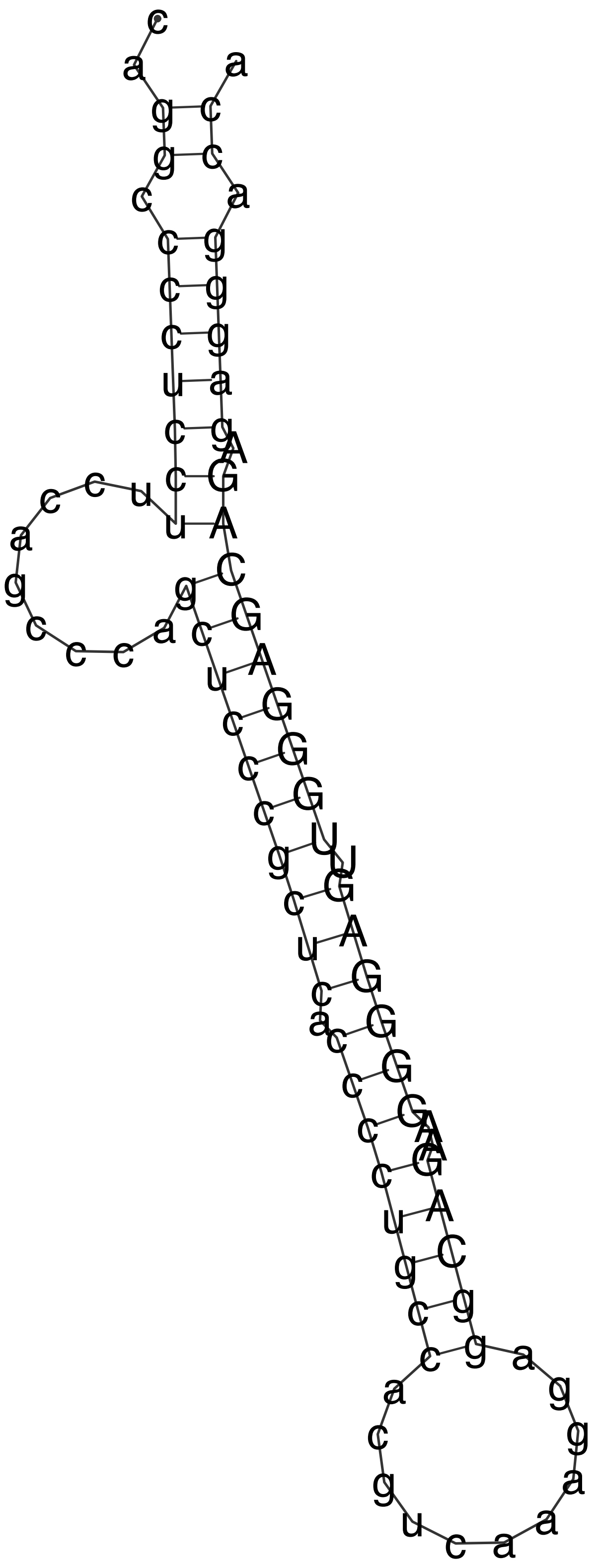

Mature sequence:

CAGAAGGGGAGUUGGGAGCAGA

Precursor sequence:

caggccccuccuuccagcccagcucccgcucaccccugccacgucaaaggaggCAGAAGGGGAGUUGGGAGCAGAgagggacca

hsa-miR-3607-3p

Genome:

APMK

Location:

4cea2|gi|530120794|gb|
APMK01023859.1|:
3672-3750_Cricetulus_gris
eus_strain_17A/
GY_chromosome_2_chr2_cont
ig_40957_whole_genome_sho
tgun_sequence

Mature sequence:

ACUGUAAACGCUUUCUGAUG

Precursor sequence:

caagguuuugguacaugugaugaag
caaaucaguaugaaaaacucaugau
ACUGUAAACGCUUUCUGAUGuauca
aacu

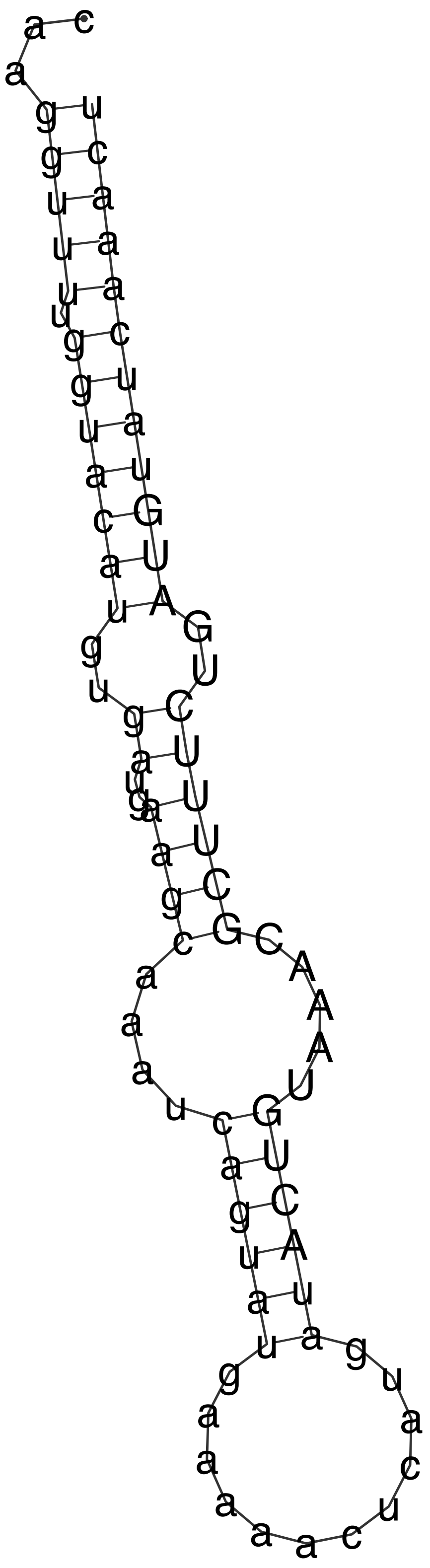

hsa-miR-449a

Genome:

APMK

Location:

1f343|gi|530148033|gb|APMK01010507.1|:c9573-9483_Cricetulus_griseus_strain_17A/GY_chromosome_2_chr2_contig_42380_whole_genome_shotgun_sequence

Mature sequence:

UGGCAGUGUAUUGUUAGCUGGU

Precursor sequence:

cugugugugacggguUGGCAGUGUAUUGUUAGCUGGUugaauaugugaaaggcaccagcuaacaugcaacugcuauccugucgcacauaca

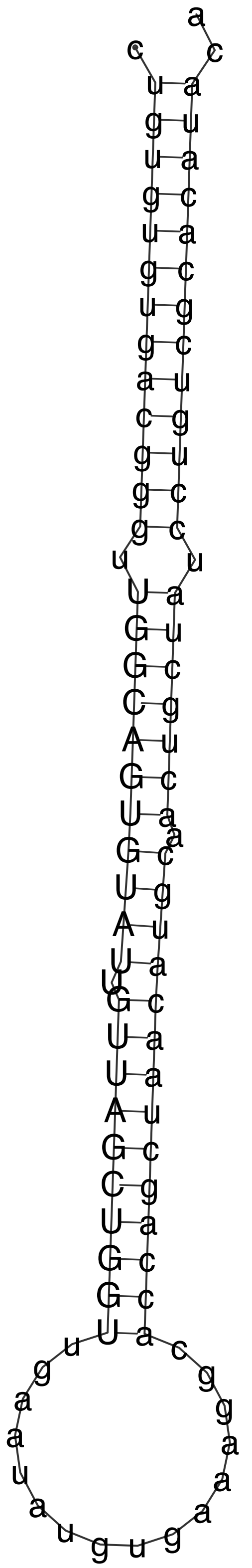

Genome:

AMDS

Location:

41d1a|gi|521767785|gb|AMDS01107287.1|:37720-37791_Cricetulus_griseus_scaffold2169_51_whole_genome_shotgun_sequence

Mature sequence:

AAACCGUUACCAUUACUGAGUU

Precursor sequence:

cuuggggauggcgaggAAACCGUUACCAUUACUGAGUUuaguaaugguaacgguucucuugcugcucccaaa

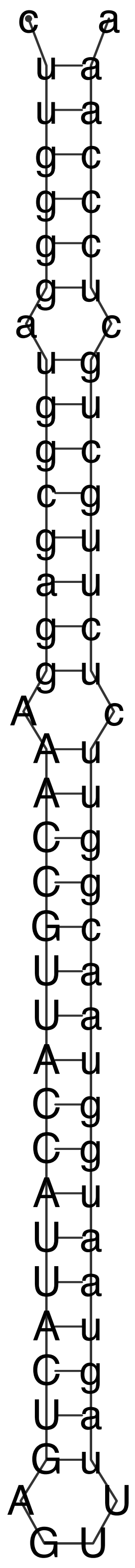

hsa-miR-574-3p

Genome:

AMDS

Location:

4f092|gi|521836155|gb|
AMDS01083733.1|:
1-84_Cricetulus_griseus_s
caffold1410_70_whole_geno
me_shotgun_sequence

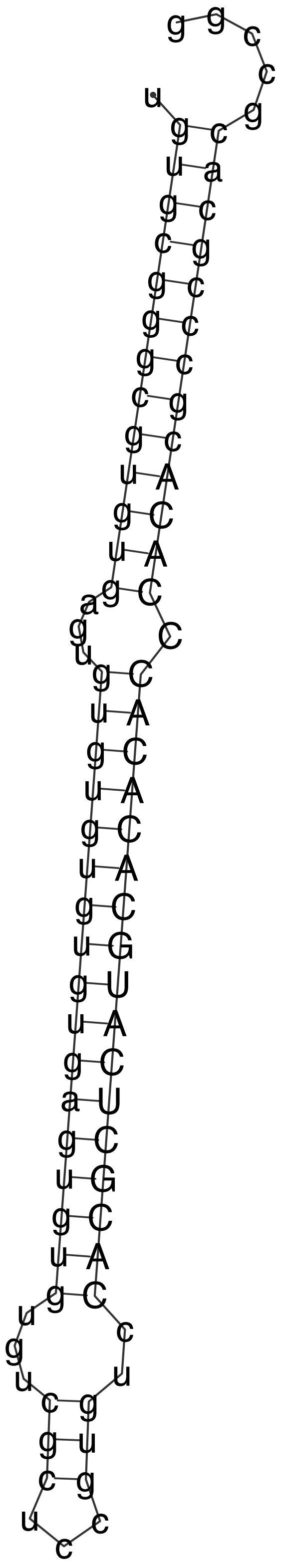

Mature sequence:

CACGCUCAUGCACACACCCACA

Precursor sequence:

ugugcgggcgugugagugugugugu
gugagugugugucgcuccgugucCA
CGCUCAUGCACACACCCACAcgccc
gcacgccgg

hsa-miR-582-5p

Genome:

APMK

Location:

79818|gi|530049938|gb|APMK01059942.1|:15470-15567_Cricetulus_griseus_strain_17A/GY_chromosome_2_chr2_contig_19771_whole_genome_shotgun_sequence

Mature sequence:

UUACAGUUGUUCAACCAGUUACU

Precursor sequence:

aucuguacucuuuggUUACAGUUGUUCAACCAGUUACUaaucuaacuaauuguaaccuguugaacaacugaacccaaagggugcaaaguuggaaacau

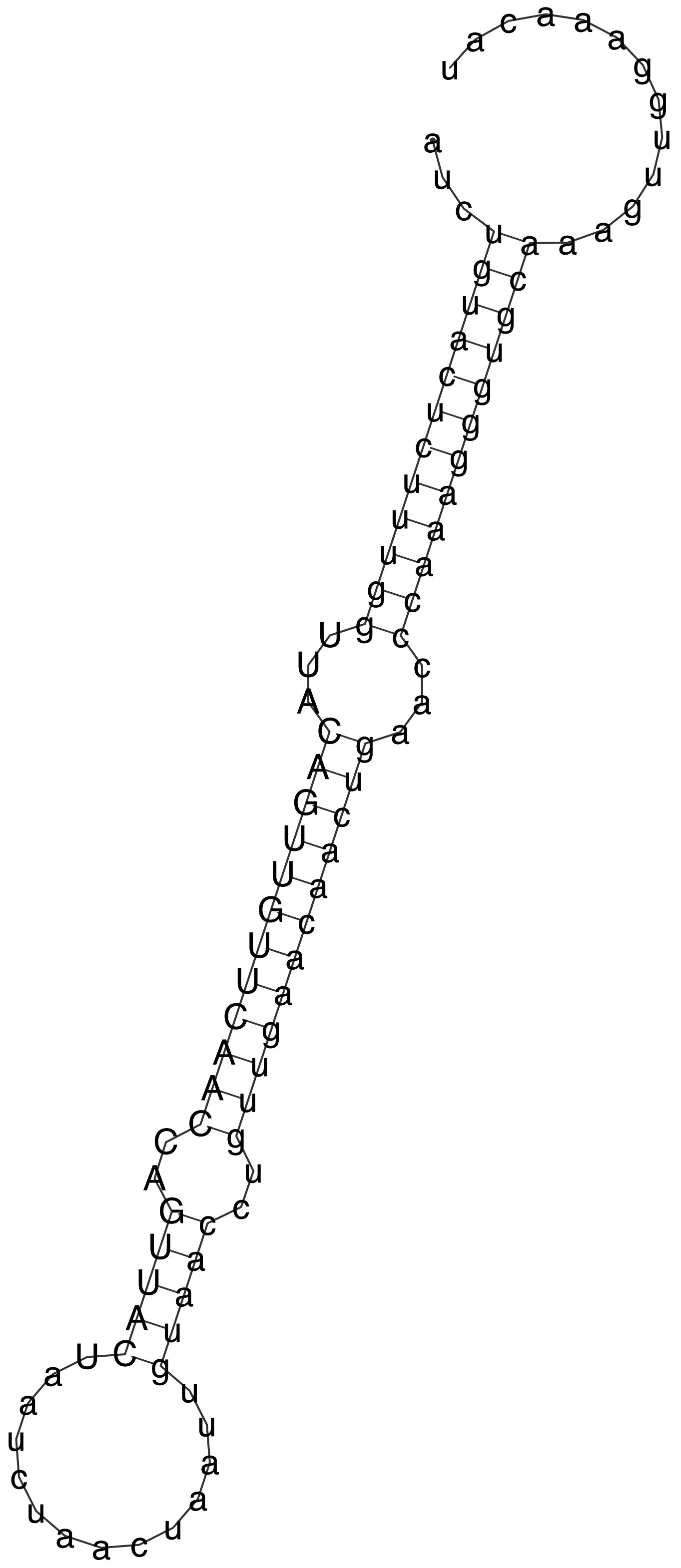

mdo-miR-125b-2-3p

Genome:

```
APMK
```

Location:

```
14bc8|gi|530059721|gb|
APMK01054927.1|:c2796-273
4_Cricetulus_griseus_stra
in_17A/
GY_chromosome_4_chr4_cont
ig_4627_whole_genome_shot
gun_sequence
```

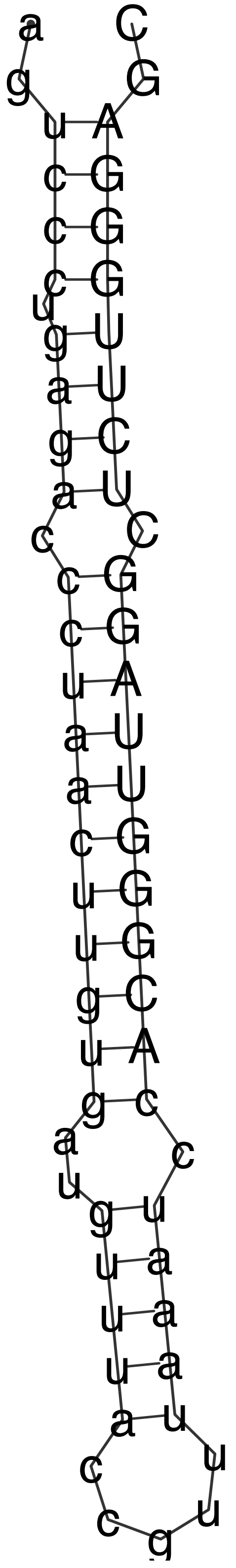

Mature sequence:

```
ACGGGUUAGGCUCUUGGGAGC
```

Precursor sequence:

```
agucccugagacccuaacuugugau
guuuaccguuuaaauccACGGGUUA
GGCUCUUGGGAGC
```

mdo-miR-29b-2-5p

Genome:

APMK

Location:

7861e|gi|529672596|gb|
APMK01239810.1|:c10108-10
045_Cricetulus_griseus_st
rain_17A/
GY_chromosome_5_chr5_cont
ig_3559_whole_genome_shot
gun_sequence

Mature sequence:

GCUGGUUUCACAUGGUGGCUUAGA

Precursor sequence:

GCUGGUUUCACAUGGUGGCUUAGAu
uuuuccaucuuuguaucuagcacca
uuugaaaucagugu

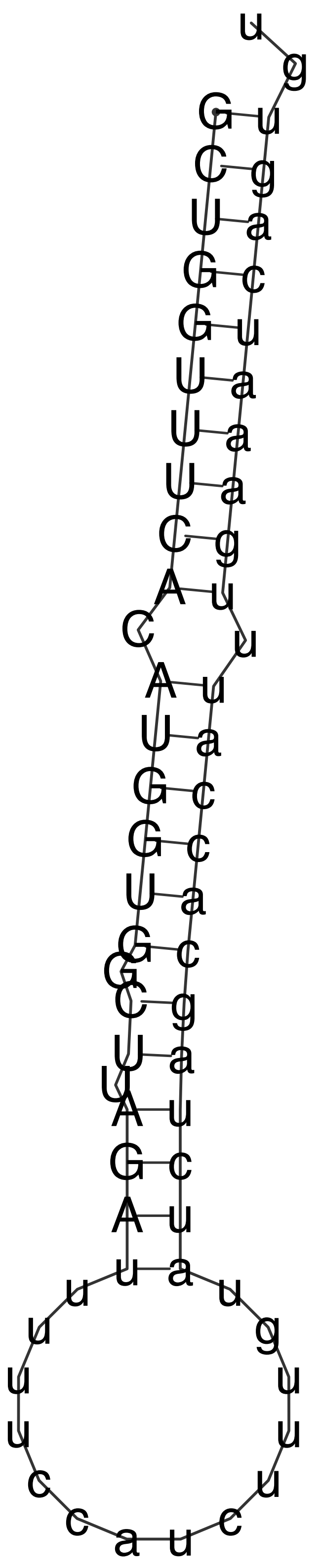

Genome:

APMK

Location:

a0830|gi|529819711|gb|
APMK01169955.1|:
9003-9090_Cricetulus_gris
eus_strain_17A/
GY_chromosome_3_chr3_cont
ig_8051_whole_genome_shot
gun_sequence

Mature sequence:

CAACUAGACUGUGAGCUUCUAGA

Precursor sequence:

gacugcccucaaggagcuuacaauc
uagcugggguaaaugacuugcacu
ugaacaCAACUAGACUGUGAGCUUC
UAGAgggcagggg

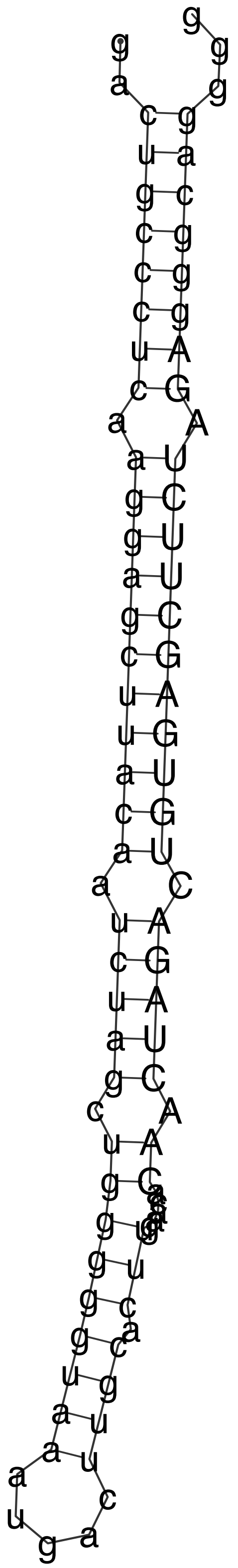

mmu-let-7a-1-3p

25a

Genome:

APMK

Location:

09164|gi|530049787|gb|
APMK01060022.1|:c20021-19
928_Cricetulus_griseus_st
rain_17A/
GY_chromosome_2_chr2_cont
ig_1047_whole_genome_shot
gun_sequence

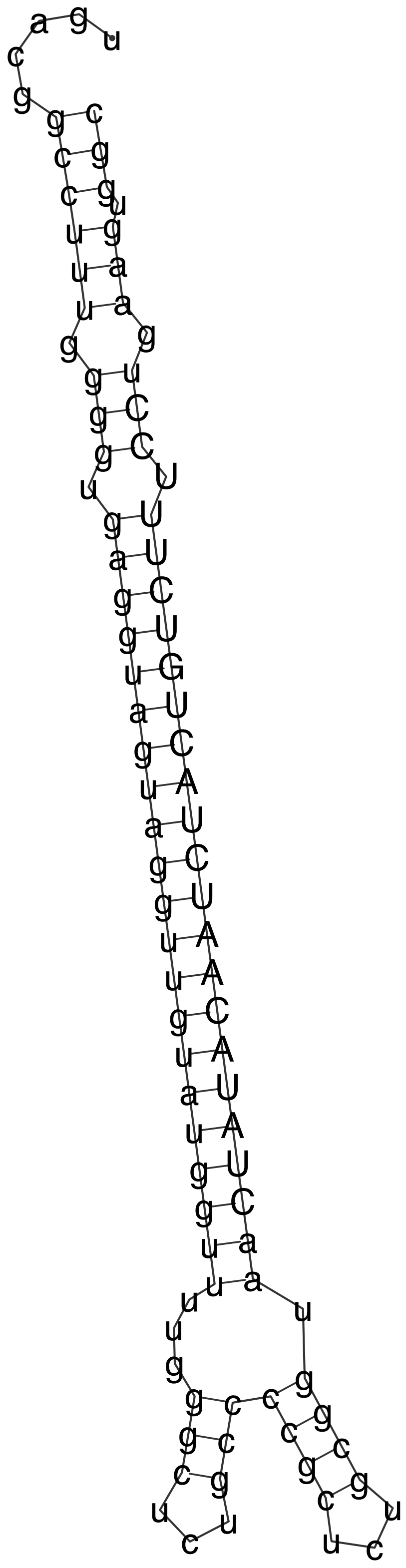

Mature sequence:

CUAUACAAUCUACUGUCUUUCC

Precursor sequence:

ugacggccuuuggggugagguagua
gguuguaugguuuugggcucugccc
cgcucugcgguaaCUAUACAAUCUA
CUGUCUUUCCugaaguggc

mmu-let-7a-1-3p

25b

Genome:

APMK

Location:

834b7|gi|529834860|gb|
APMK01162609.1|:c5514-542
1_Cricetulus_griseus_stra
in_17A/
GY_chromosome_3_chr3_cont
ig_8789_whole_genome_shot
gun_sequence

Mature sequence:

CUAUACAAUCUACUGUCUUUCC

Precursor sequence:

uucacugugggaugagguaguaggu
uguauaguuuuagggucacacccac
cacugggagauaaCUAUACAAUCUA
CUGUCUUUCCuaaggugau

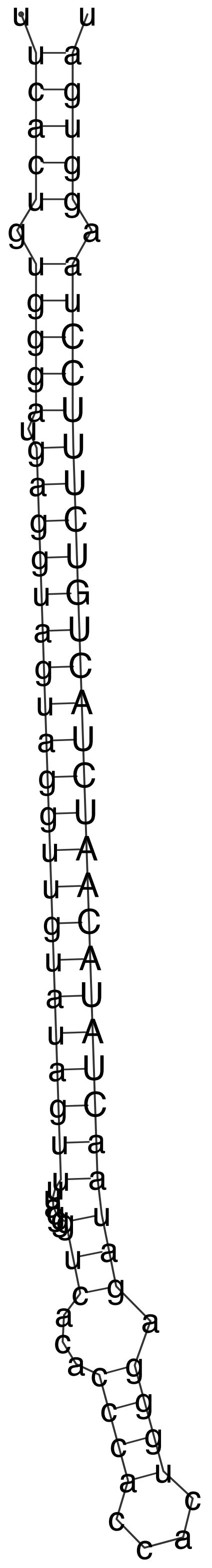

mmu-miR-101a-5p

Genome:

APMK

Location:

a1f1a|gi|529733102|gb|APMK01211335.1|:c1818-1736_Cricetulus_griseus_strain_17A/GY_chromosome_2_chr2_contig_2632_whole_genome_shotgun_sequence

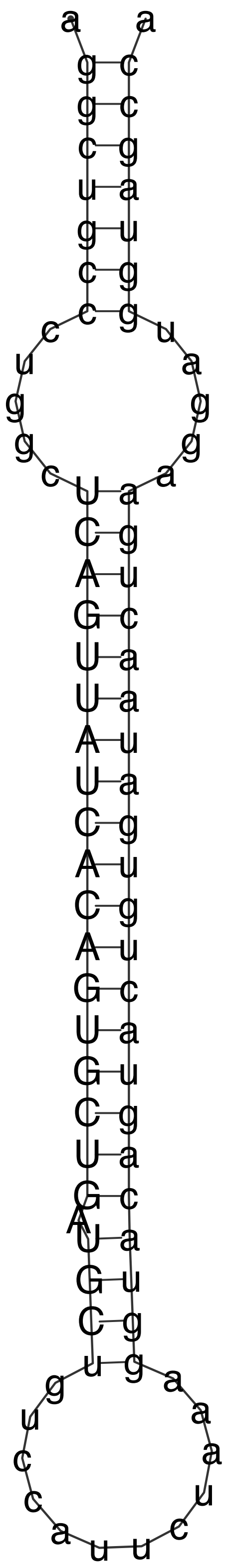

Mature sequence:

UCAGUUAUCACAGUGCUGAUGC

Precursor sequence:

aggcugcccuggcUCAGUUAUCACAGUGCUGAUGCuguccauucuaaagguacaguacugugauaacugaaggauggguagcca

mmu-miR-106b-5p

Genome:

APMK

Location:

dfc31|gi|530066320|gb|
APMK01051712.1|:
4526-4607_Cricetulus_gris
eus_strain_17A/
GY_chromosome_4_chr4_cont
ig_33445_whole_genome_sho
tgun_sequence

Mature sequence:

UAAAGUGCUGACAGUGCAGAU

Precursor sequence:

ccugcuggggcUAAAGUGCUGACAG
UGCAGAUagugguccucucugugcu
accgcacugugggguacuugcugcuc
cagcagg

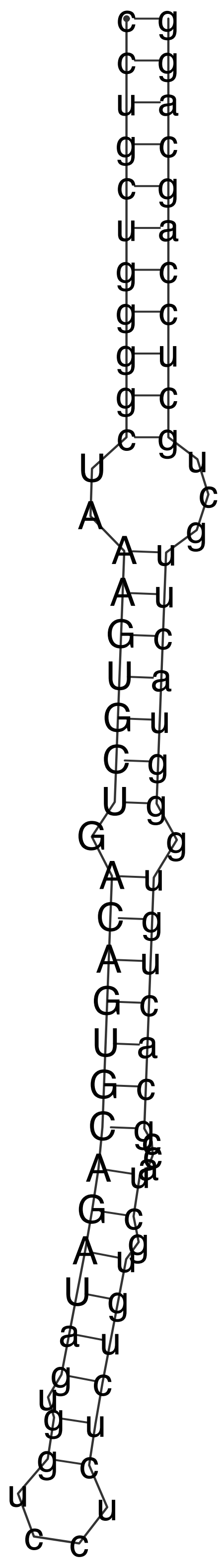

mmu-miR-107-5p

Genome:

APMK

Location:

41ad3|gi|529518969|gb|APMK01310302.1|:c15036-14950_Cricetulus_griseus_strain_17A/GY_chromosome_3_chr3_contig_22832_whole_genome_shotgun_sequence

Mature sequence:

AGCUUCUUUACAGUGUUGCCUUG

Precursor sequence:

uucucucugcuuucAGCUUCUUUACAGUGUUGCCUUGuggcauggaauucaagcagcauuguacagggcuaucaaagcacugagagc

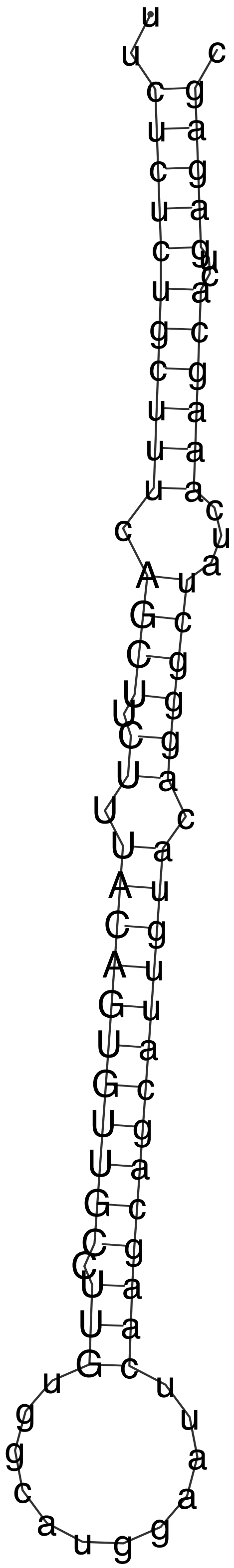

mmu-miR-1224-5p

Genome:

K1-BB

Location:

82668|lcl|
NODE_9612279_length_106_c
ov_1.000000:c87-3

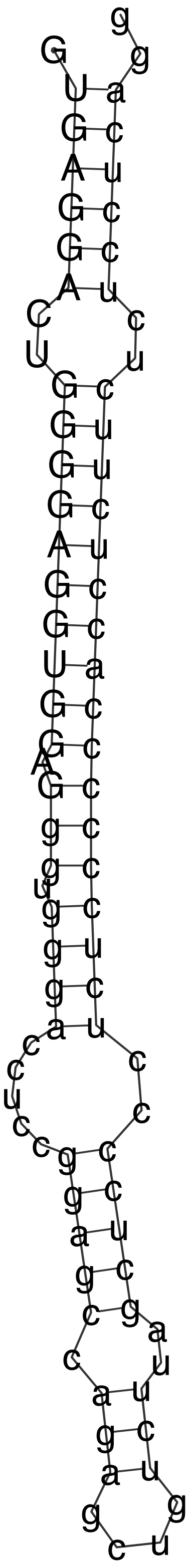

Mature sequence:

GUGAGGACUGGGGAGGUGGAG

Precursor sequence:

GUGAGGACUGGGGAGGUGGAGggug
ggaccuccggagccagagcugucuu
agcuccccucuccccccaccucuuc
ucuccucagg

mmu-miR-1249-3p

Genome:

APMK

Location:

d98b9|gi|529756299|gb|
APMK01199882.1|:
3446-3543_Cricetulus_gris
eus_strain_17A/
GY_chromosome_2_chr2_cont
ig_1116_whole_genome_shot
gun_sequence

Mature sequence:

ACGCCCUUCCCCCCCUUCUUCA

Precursor sequence:

cgauucuuguuugcauggggaggag
ggaggggaugggccaaguucccugu
gccuggaACGCCCUUCCCCCCCUUC
UUCAccuggcgaacuccuacucg

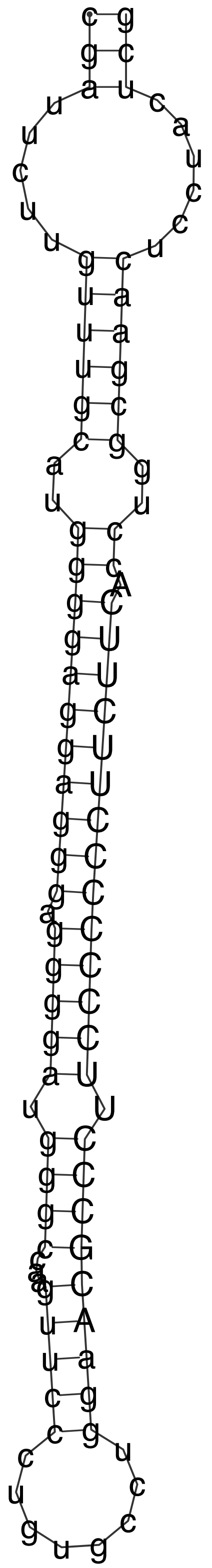

mmu-miR-126a-5p

Genome:

APMK

Location:

69d6b|gi|529961444|gb|APMK01099990.1|:c12754-12682_Cricetulus_griseus_strain_17A/GY_chromosome_6_chr6_contig_14697_whole_genome_shotgun_sequence

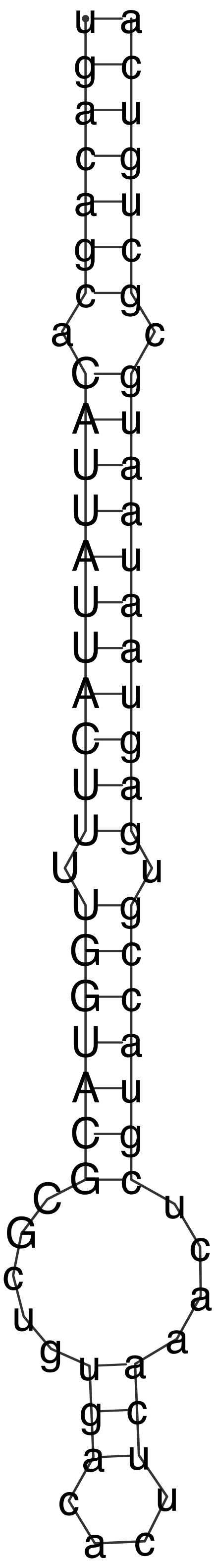

Mature sequence:

CAUUAUUACUUUUGGUACGCG

Precursor sequence:

ugacagcaCAUUAUUACUUUUGGUACGCGcugugacacuucaaacucguaccgugaguaauaaugcgcuguca

mmu-miR-126b-3p

Genome:

```
APMK
```

Location:

```
c9ccf|gi|529961444|gb|
APMK01099990.1|:
12691-12748_Cricetulus_gr
iseus_strain_17A/
GY_chromosome_6_chr6_cont
ig_14697_whole_genome_sho
tgun_sequence
```

Mature sequence:

```
CGCGUACCAAAAGUAAUAAUGUG
```

Precursor sequence:

```
auuauuacucacgguacgaguuuga
agugucacagCGCGUACCAAAAGUA
AUAAUGUG
```

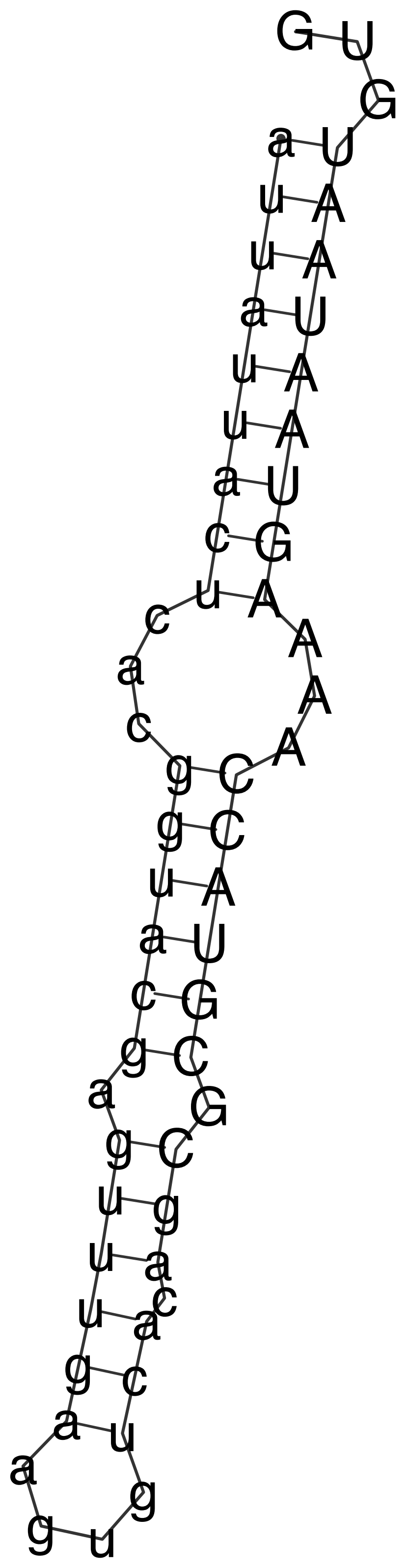

mmu-miR-127-3p

Genome:

APMK

Location:

e9090|gi|529938472|gb|APMK01110536.1|:c14656-14587_Cricetulus_griseus_strain_17A/GY_chromosome_5_chr5_contig_27034_whole_genome_shotgun_sequence

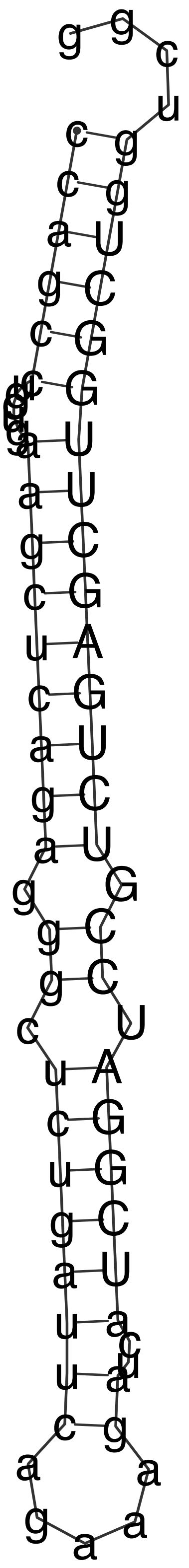

Mature sequence:

UCGGAUCCGUCUGAGCUUGGCU

Precursor sequence:

ccagccugcugaagcucagagggcucugauucagaaagaucaUCGGAUCCGUCUGAGCUUGGCUggucgg

Genome:

APMK

Location:

df834|gi|530047046|gb|APMK01061418.1|:c7194-7130_Cricetulus_griseus_strain_17A/GY_chromosome_6_chr6_contig_18801_whole_genome_shotgun_sequence

Mature sequence:

UCUCCCAACCCUUGUACCAGUG

Precursor sequence:

cccugUCUCCCAACCCUUGUACCAGUGcugcacuucagacccugguacaggccugggggcaggg

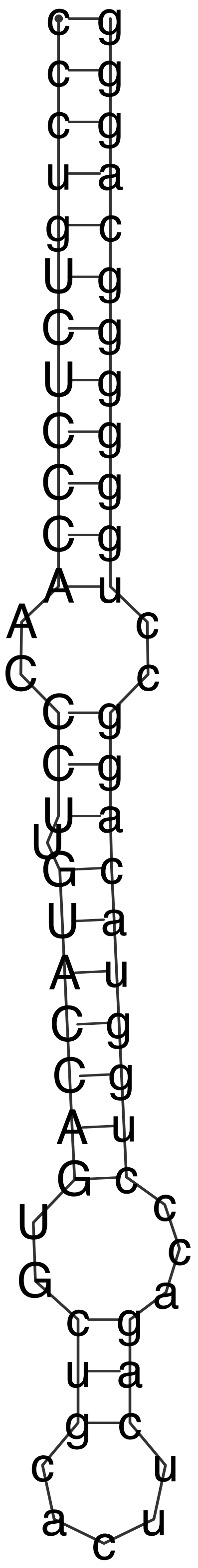

mmu-miR-16-2-3p

Genome:

APMK

Location:

2f19f|gi|529819616|gb|
APMK01169993.1|:
18730-18824_Cricetulus_gr
iseus_strain_17A/
GY_chromosome_1_chr1_cont
ig_9886_whole_genome_shot
gun_sequence

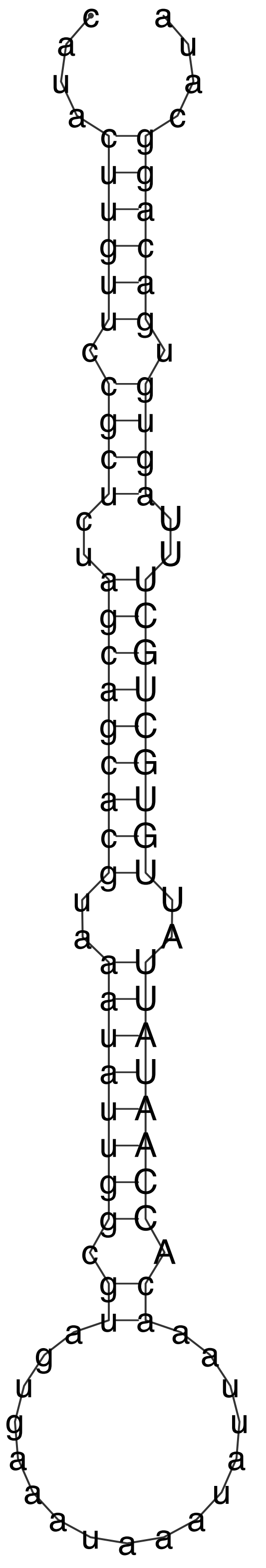

Mature sequence:

ACCAAUAUUAUUGUGCUGCUUU

Precursor sequence:

cauacuuguuccgcucuagcagcac
guaaauauuggcguagugaaauaaa
uauuaaacACCAAUAUUAUUGUGCU
GCUUUagugugacaggcaua

mmu-miR-1843a-5p

Genome:

APMK

Location:

bfda0|gi|529576210|gb|APMK01282820.1|:c2473-2409_Cricetulus_griseus_strain_17A/GY_chromosome_5_chr5_contig_4711_whole_genome_shotgun_sequence

Mature sequence:

UAUGGAGGUCUCUGUCUGACU

Precursor sequence:

gaaUAUGGAGGUCUCUGUCUGACUuagggcagcuggcuaagucugaucguucaccuccauacaag

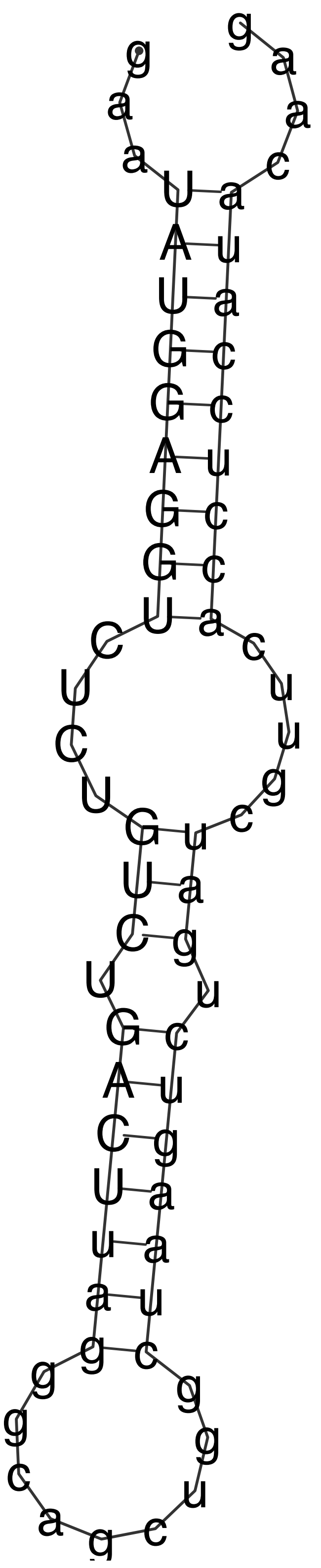

mmu-miR-187-5p

Genome:

APMK

Location:

d8a38|gi|529652815|gb|
APMK01249572.1|:c15322-15
262_Cricetulus_griseus_st
rain_17A/
GY_chromosome_2_chr2_cont
ig_17135_whole_genome_sho
tgun_sequence

Mature sequence:

AGGCUACAACACAGGACCCGGG

Precursor sequence:

ucAGGCUACAACACAGGACCCGGGc
gcuccucugaccccucgugucuugu
guugcagccgg

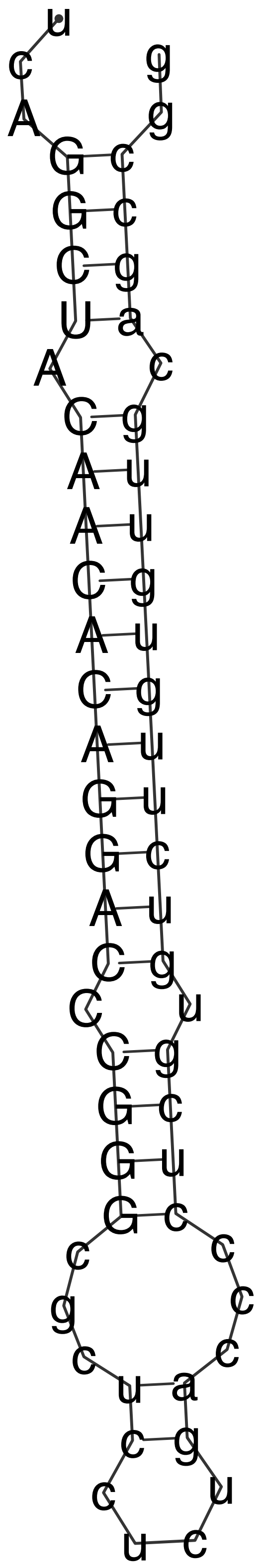

mmu-miR-190a-3p

Genome:

APMK

Location:

9025c|gi|530020316|gb|
APMK01074755.1|:
2396-2462_Cricetulus_gris
eus_strain_17A/
GY_chromosome_4_chr4_cont
ig_20537_whole_genome_sho
tgun_sequence

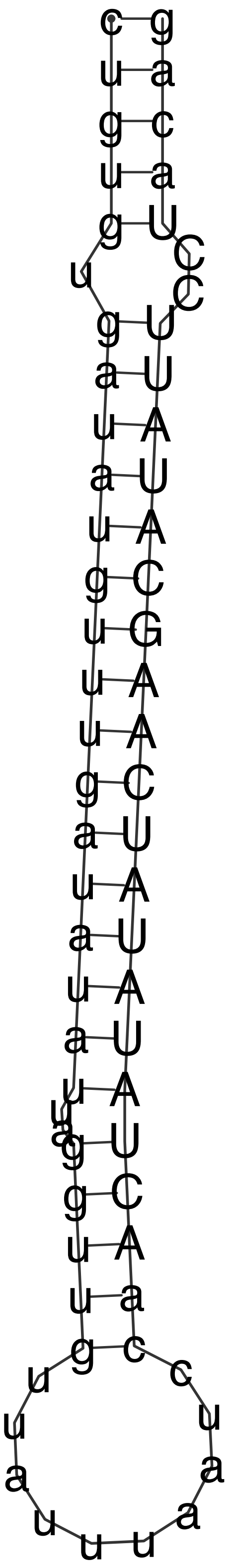

Mature sequence:

ACUAUAUAUCAAGCAUAUUCCU

Precursor sequence:

cugugugauauguuugauauauuag
guuguuauuuaauccaACUAUAUAU
CAAGCAUAUUCCUacag

mmu-miR-193a-3p

Genome:

AMDS

Location:

9d8f0|gi|521804602|gb|
AMDS01094291.1|:
17-82_Cricetulus_griseus_
scaffold1795_15_whole_gen
ome_shotgun_sequence

Mature sequence:

AACUGGCCUACAAAGUCCCAGU

Precursor sequence:

gagagcugggucuuugcgggcaaga
ugagggugucaguucAACUGGCCUA
CAAAGUCCCAGUccuc

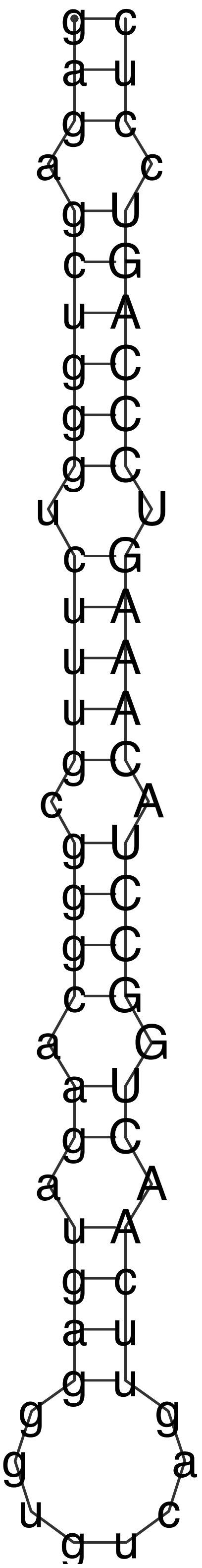

Genome:

AMDS

Location:

cad29|gi|521804602|gb|AMDS01094291.1|:17-82_Cricetulus_griseus_scaffold1795_15_whole_genome_shotgun_sequence

Mature sequence:

UGGGUCUUUGCGGGCAAGAUGA

Precursor sequence:

gagagcUGGGUCUUUGCGGGCAAGAUGAgggugucaguucaacuggccuacaaagucccaguccuc

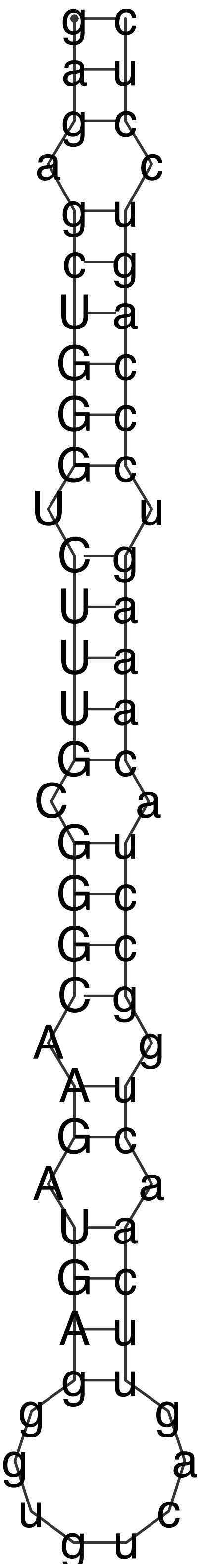

mmu-miR-1983

Genome:

APMK

Location:

8a7d9|gi|529554673|gb|
APMK01293597.1|:c4384-425
3_Cricetulus_griseus_stra
in_17A/
GY_chromosome_3_chr3_cont
ig_26483_whole_genome_sho
tgun_sequence

Mature sequence:

CUCACCUGGAGCAUGUUUUCU

Precursor sequence:

ccaucacagagaaagcaugcuccag
uggcgcaaucgguuagcgcgcggua
cuuauacagcaguauaugugcgggu
gaugccgagguugugaguucgagcC
UCACCUGGAGCAUGUUUUCUucccu
uuccggu

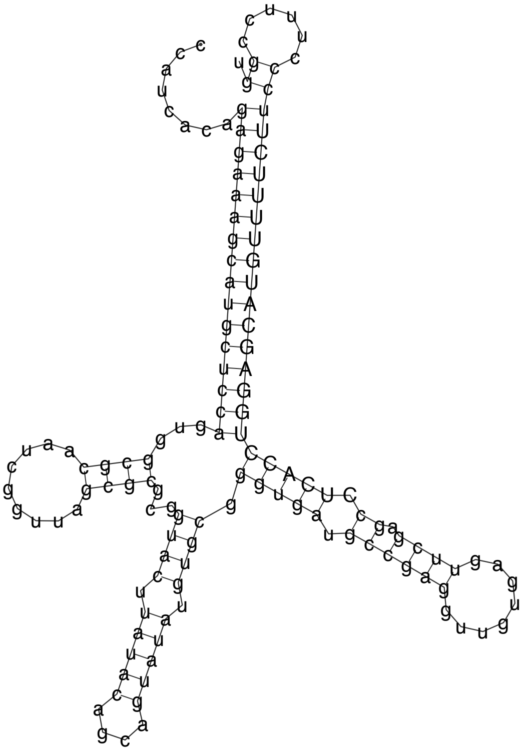

mmu-miR-199a-5p

Genome:

APMK

Location:

32228|gi|530169397|gb|APMK01000004.1|:15047-15156_Cricetulus_griseus_strain_17A/GY_chromosome_5_chr5_contig_3918_whole_genome_shotgun_sequence

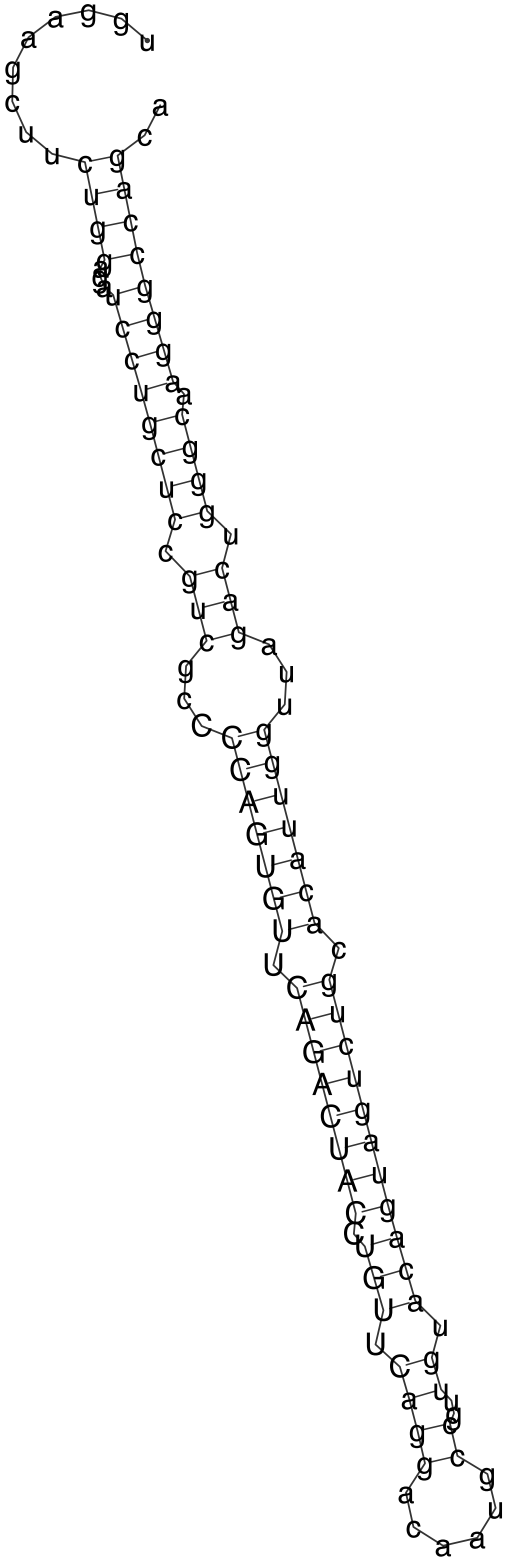

Mature sequence:

CCCAGUGUUCAGACUACCUGUUC

Precursor sequence:

uggaagcuucuggagauccugcuccgucgcCCCAGUGUUCAGACUACCUGUUCaggacaaugccguuguacaguagucugcacauugguuagacugggcaagggccagca

Genome:

APMK

Location:

bf127|gi|530128314|gb|APMK01020406.1|:16017-16138_Cricetulus_griseus_strain_17A/GY_chromosome_2_chr2_contig_24928_whole_genome_shotgun_sequence

Mature sequence:

UGGGUACAUAAAGAAGUAUGUGC

Precursor sequence:

gaaagcauugcuccucuuggugugccauuaccuacccaaaauacguacuucuuuacauuccauagcaguguauguucauaUGGGUACAUAAAGAAGUAUGUGCucugaguaggcacugcugag

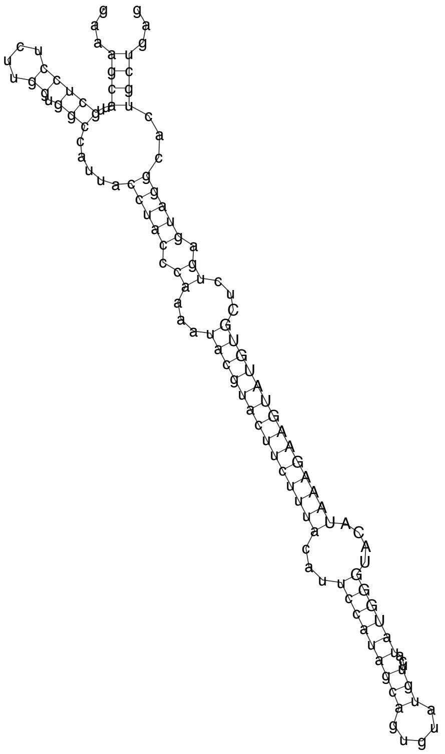

Genome:

APMK

Location:

0b0c3|gi|529730011|gb|
APMK01212857.1|:c1138-106
7_Cricetulus_griseus_stra
in_17A/
GY_chromosome_3_chr3_cont
ig_11248_whole_genome_sho
tgun_sequence

Mature sequence:

UUCCUAUGCAUAUACUUCUUU

Precursor sequence:

guuccuuuUUCCUAUGCAUAUACUU
CUUUguggaacuggucuaaagaggu
guagugcaugggaaaauggagc

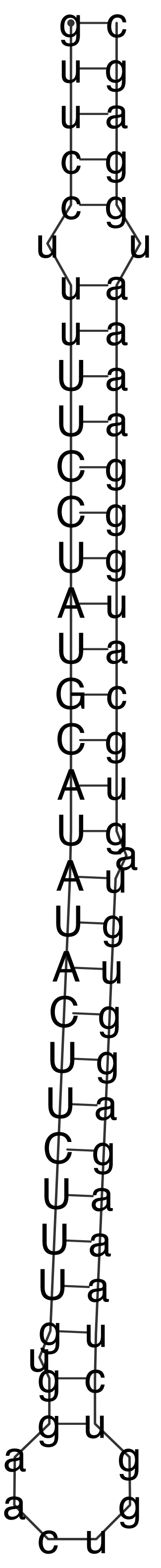

Genome:

APMK

Location:

34636|gi|529982091|gb|APMK01090750.1|:c5608-5503_Cricetulus_griseus_strain_17A/GY_chromosome_3_chr3_contig_20497_whole_genome_shotgun_sequence

Mature sequence:

UUCCCUUUGUCAUCCUUUGCCU

Precursor sequence:

cuaccuagaccagugacuugugggcUUCCCUUUGUCAUCCUUUGCCUaggccucugaguggggcaaggacagcaaaggggggcucaguggucaccucuacugcaga

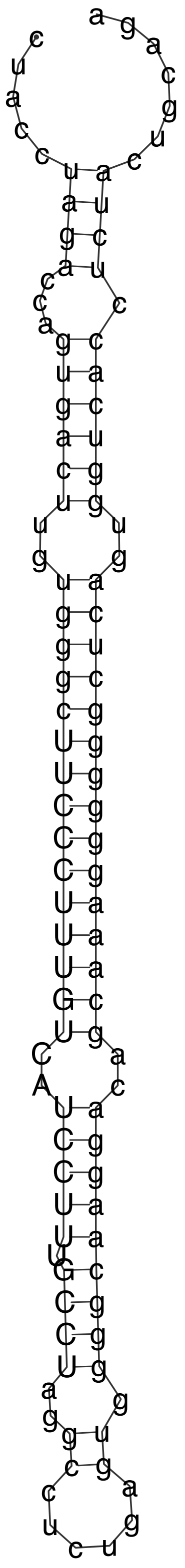

mmu-miR-216b-5p

Genome:

APMK

Location:

87245|gi|529560949|gb|
APMK01290466.1|:
3324-3409_Cricetulus_gris
eus_strain_17A/
GY_chromosome_1_chr1_cont
ig_50994_whole_genome_sho
tgun_sequence

Mature sequence:

AAAUCUCUGCAGGCAAAUGUGA

Precursor sequence:

guggcagacugagAAAUCUCUGCAG
GCAAAUGUGAugucagugaagaaau
cacacacuuaccuguagagauucuu
cagucugucaa

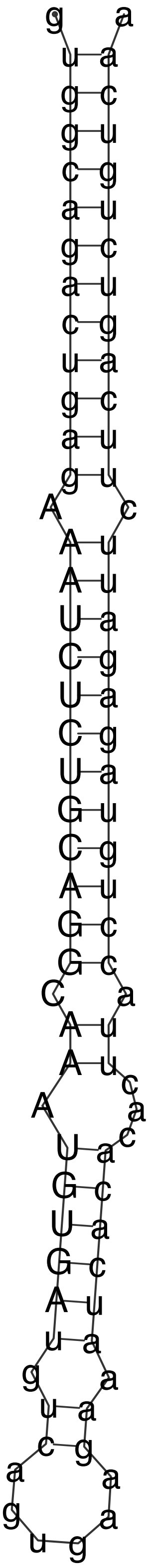

mmu-miR-24-1-5p

Genome:

APMK

Location:

ff283|gi|530144123|gb|
APMK01012466.1|:c4774-470
7_Cricetulus_griseus_stra
in_17A/
GY_chromosome_3_chr3_cont
ig_19203_whole_genome_sho
tgun_sequence

Mature sequence:

GUGCCUACUGAGCUGAUAUCAGU

Precursor sequence:

cuccgGUGCCUACUGAGCUGAUAUC
AGUucucauuucacacacuggcuca
guucagcaggaacaggag

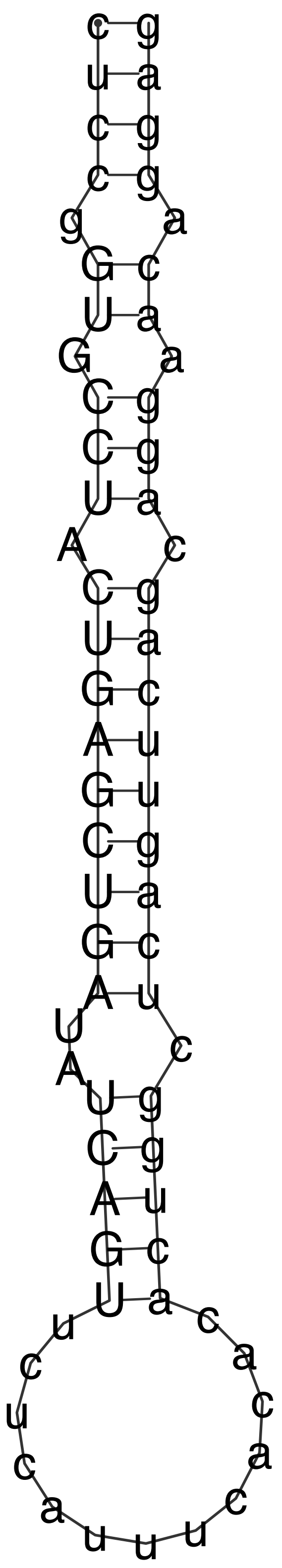

Genome:

APMK

Location:

4a2cc|gi|530140384|gb|
APMK01014396.1|:c1077-999
_Cricetulus_griseus_strai
n_17A/
GY_chromosome_6_chr6_cont
ig_24005_whole_genome_sho
tgun_sequence

Mature sequence:

AGGGCCCCCCCUCAAUCCUGU

Precursor sequence:

gggccuuucuggAGGGCCCCCCCUC
AAUCCUGUugugcucgcuucagagg
guugggguggaggcucuccugaagga
gucu

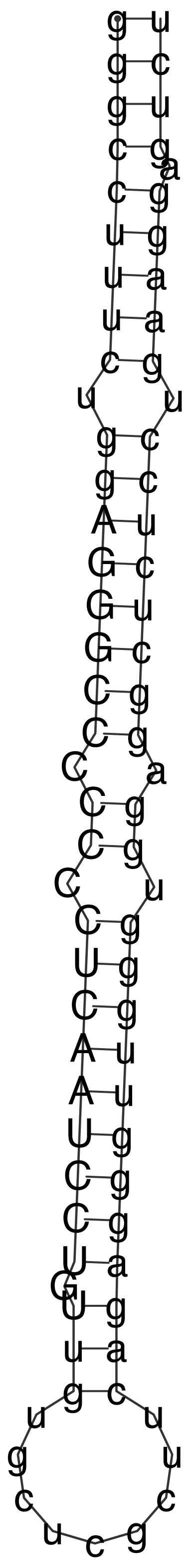

mmu-miR-3068-3p

Genome:

APMK

Location:

df7ea|gi|529588245|gb|
APMK01280478.1|:
7705-7783_Cricetulus_gris
eus_strain_17A/
GY_chromosome_5_chr5_cont
ig_3429_whole_genome_shot
gun_sequence

Mature sequence:

GGUGAAUUGCAGUACUCCAACA

Precursor sequence:

gcgagaguaauuggaguucaugcaa
guucuaaccagcuuaaucaguagcu
gGGUGAAUUGCAGUACUCCAACAuu
cugu

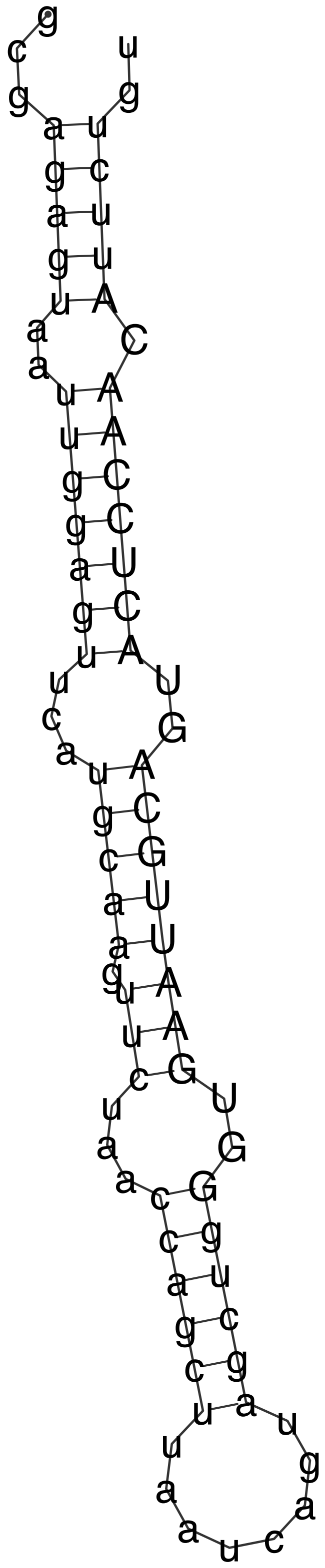

Genome:

APMK

Location:

ab9fa|gi|529588245|gb|APMK01280478.1|:7705-7783_Cricetulus_griseus_strain_17A/GY_chromosome_5_chr5_contig_3429_whole_genome_shotgun_sequence

Mature sequence:

UUGGAGUUCAUGCAAGUUCUAACC

Precursor sequence:

gcgagaguaaUUGGAGUUCAUGCAAGUUCUAACCagcuuaaucaguagcugggugaauugcaguacuccaacauucugu

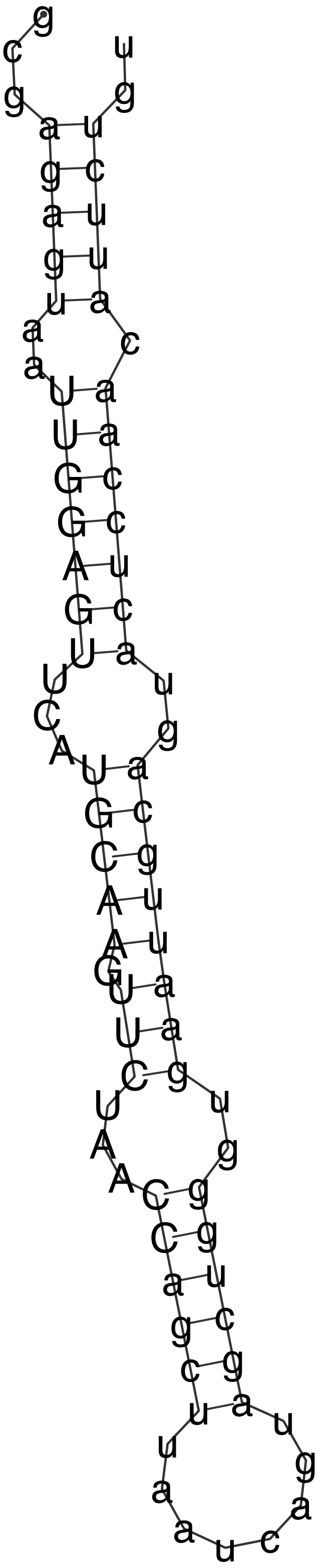

mmu-miR-3074-5p

Genome:

APMK

Location:

33d99|gi|529810626|gb|APMK01174175.1|:c2471-2389_Cricetulus_griseus_strain_17A/GY_chromosome_3_chr3_contig_31791_whole_genome_shotgun_sequence

Mature sequence:

GUUCCUGCUGAACUGAGCCAGU

Precursor sequence:

gggcuggacuccuGUUCCUGCUGAACUGAGCCAGUgcacuggaaucaacuguuucagcucaguaggcacaggaggcggagccc

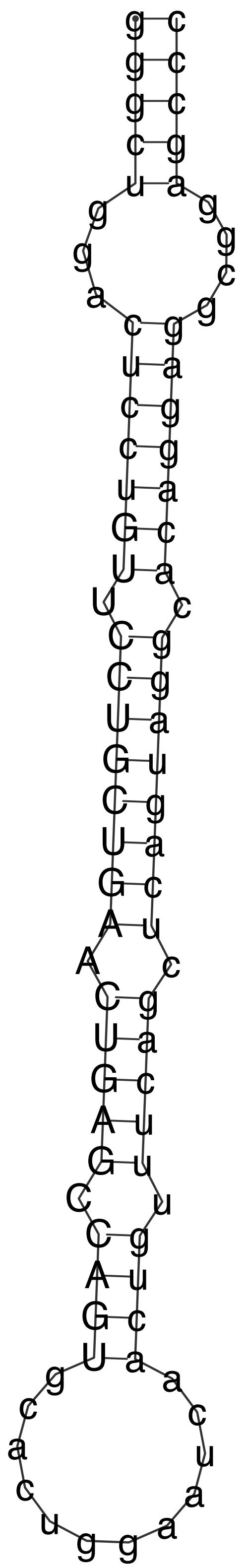

mmu-miR-3074-5p

51b

Genome:

APMK

Location:

585ac|gi|530144123|gb|
APMK01012466.1|:
4699-4781_Cricetulus_gris
eus_strain_17A/
GY_chromosome_3_chr3_cont
ig_19203_whole_genome_sho
tgun_sequence

Mature sequence:

GUUCCUGCUGAACUGAGCCAGU

Precursor sequence:

gggcucgacuccuGUUCCUGCUGAA
CUGAGCCAGUgugugaaaugagaac
ugauaucagcucaguaggcaccgga
gggcgggu

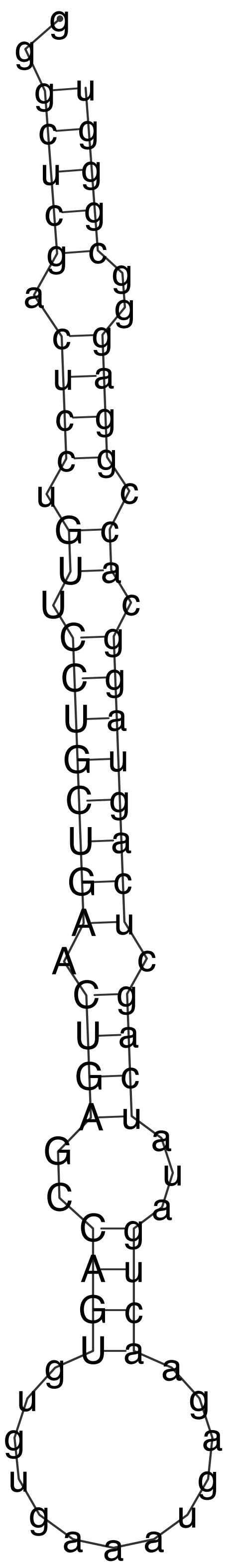

mmu-miR-30c-1-3p

Genome:

APMK

Location:

906ab|gi|529671265|gb|
APMK01240434.1|:
7851-7939_Cricetulus_gris
eus_strain_17A/
GY_chromosome_2_chr2_cont
ig_12921_whole_genome_sho
tgun_sequence

Mature sequence:

CUGGGAGAGGGUUGUUUACUCC

Precursor sequence:

accauguuguaguguguguaaacau
ccuacacucucagcugugagcucaa
gguggCUGGGAGAGGGUUGUUUACU
CCuucugccaugga

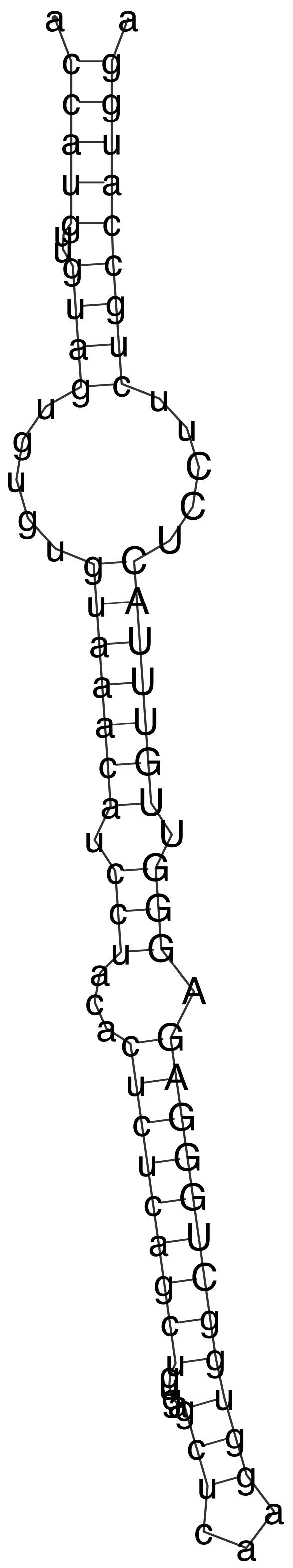

mmu-miR-3102-3p

Genome:

```
APMK
```

Location:

```
261cd|gi|529776827|gb|
APMK01190477.1|:c4467-436
4_Cricetulus_griseus_stra
in_17A/
GY_chromosome_3_chr3_cont
ig_8683_whole_genome_shot
gun_sequence
```

Mature sequence:

```
GAGCACCCCAUUGGCUACCCACA
```

Precursor sequence:

```
gugaguggccagggugggggcugggu
ggugcaggcaggagagccauugauc
uauugugggcucuacucccugcccc
agccaGAGCACCCCAUUGGCUACCC
ACAg
```

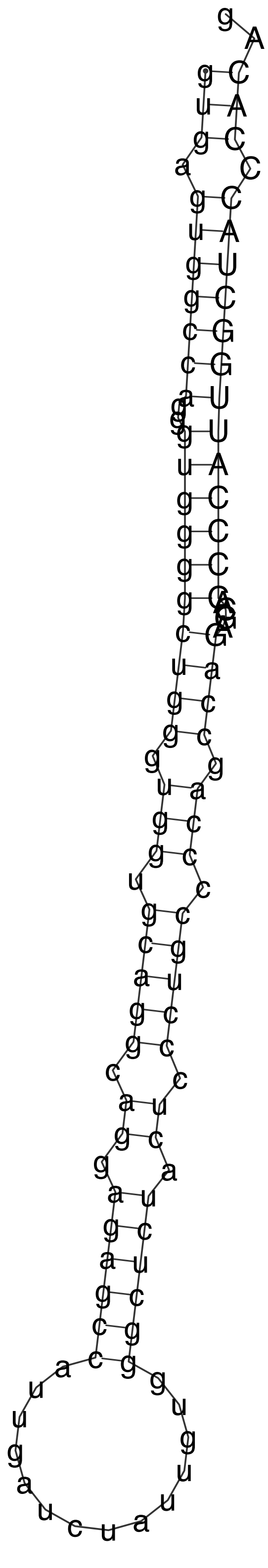

mmu-miR-3102-5p.2-5p 54

Genome:

APMK

Location:

85e01|gi|529776827|gb|
APMK01190477.1|:c4467-436
4_Cricetulus_griseus_stra
in_17A/
GY_chromosome_3_chr3_cont
ig_8683_whole_genome_shot
gun_sequence

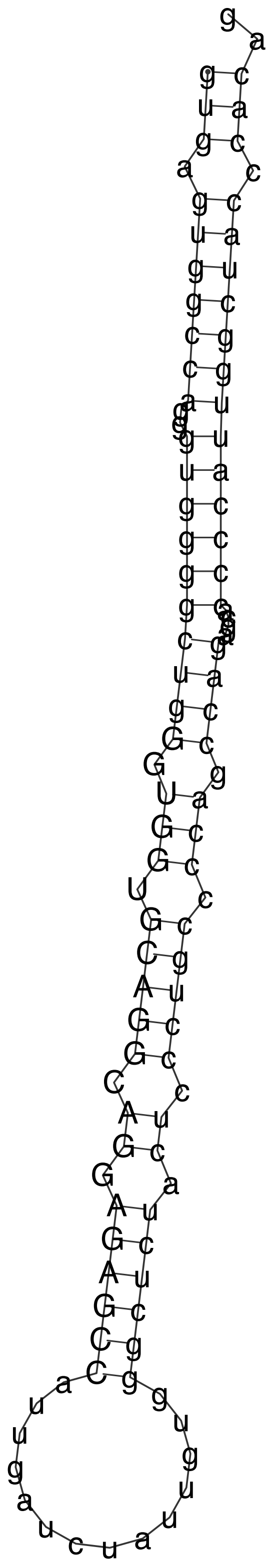

Mature sequence:

GGUGGUGCAGGCAGGAGAGCC

Precursor sequence:

gugaguggccaggguggggcugGGU
GGUGCAGGCAGGAGAGCCauugauc
uauugugggcucuacucccugcccc
agccagagcaccccauuggcuaccc
acag

mmu-miR-320-5p

Genome:

APMK

Location:

abe95|gi|529631796|gb|
APMK01259908.1|:
19209-19290_Cricetulus_gr
iseus_strain_17A/
GY_chromosome_1_chr1_cont
ig_26577_whole_genome_sho
tgun_sequence

Mature sequence:

GCCUUCUCUUCCCGGUUCUUCC

Precursor sequence:

gccucgcuacccuccGCCUUCUCUU
CCCGGUUCUUCCcggagucgggaaa
agcugggguugagagggcgaaaaaaa
aaggaug

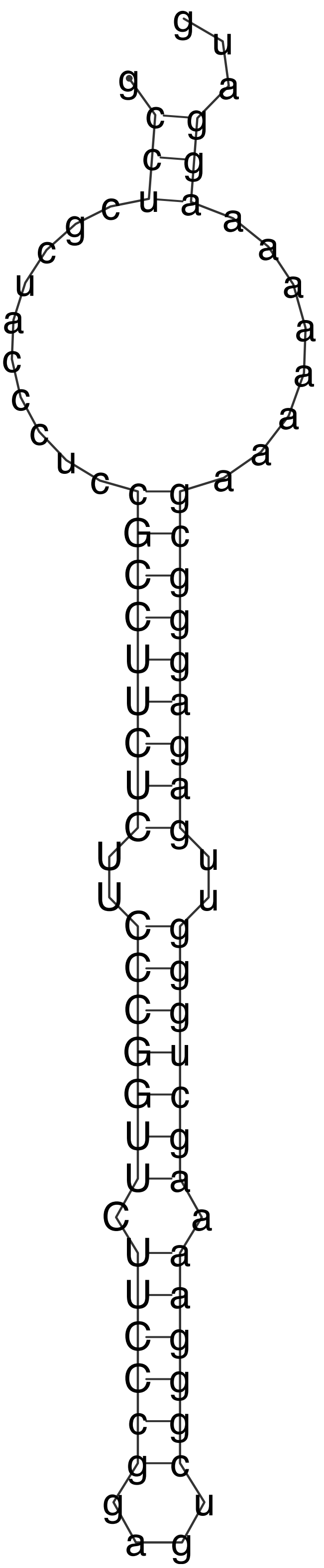

mmu-miR-328-5p

Genome:

APMK

Location:

641e0|gi|529984358|gb|
APMK01089955.1|:
11435-11531_Cricetulus_gr
iseus_strain_17A/
GY_chromosome_3_chr3_cont
ig_15613_whole_genome_sho
tgun_sequence

Mature sequence:

GGGGGGCAGGAGGGGCUCAGGG

Precursor sequence:

cugucucggagccuggagcgGGGGG
GCAGGAGGGGCUCAGGGagaaagug
ucuacagccccuggcccucucugcc
cuuccguccccugacuccaaau

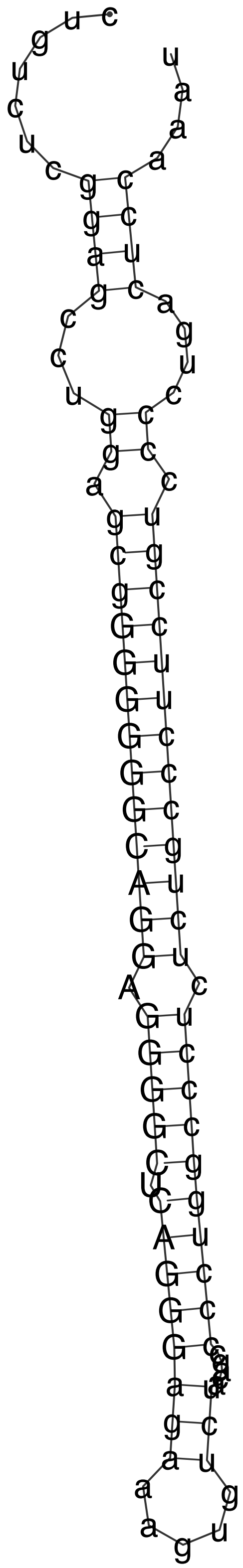

Genome:

APMK

Location:

fa415|gi|529815370|gb|APMK01171942.1|:991-1137_Cricetulus_griseus_strain_17A/GY_chromosome_2_chr2_contig_24151_whole_genome_shotgun_sequence

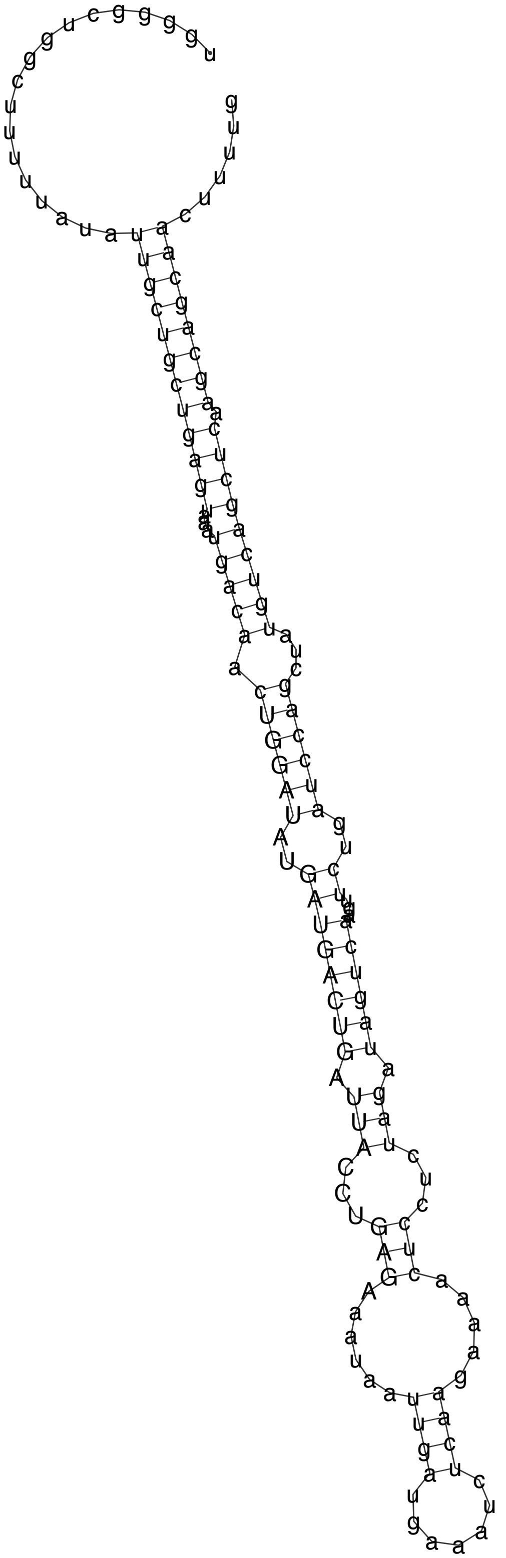

Mature sequence:

UGGAUAUGAUGACUGAUUACCUGAGA

Precursor sequence:

uggggcuggcuuuuuauauugcugcugaguaaaugacaacUGGAUAUGAUGACUGAUUACCUGAGAaauaauugaugaaaucucaagaaaacuccucuagauagucaaguucugauccagcuaugucagcucaagcagcaacuuuug

mmu-miR-365-2-5p

Genome:

APMK

Location:

93475|gi|530068699|gb|APMK01050533.1|:c15009-14898_Cricetulus_griseus_strain_17A/GY_chromosome_7_chr7_contig_5275_whole_genome_shotgun_sequence

Mature sequence:

AGGGACUUUCAGGGGCAGCUGUG

Precursor sequence:

cgcauguucaaggacagcaagaaaa
augAGGGACUUUCAGGGGCAGCUGU
Guuuucugacucagucauaaugccc
cuaaaaauccuuauuguucuugcag
ugugcaucaggc

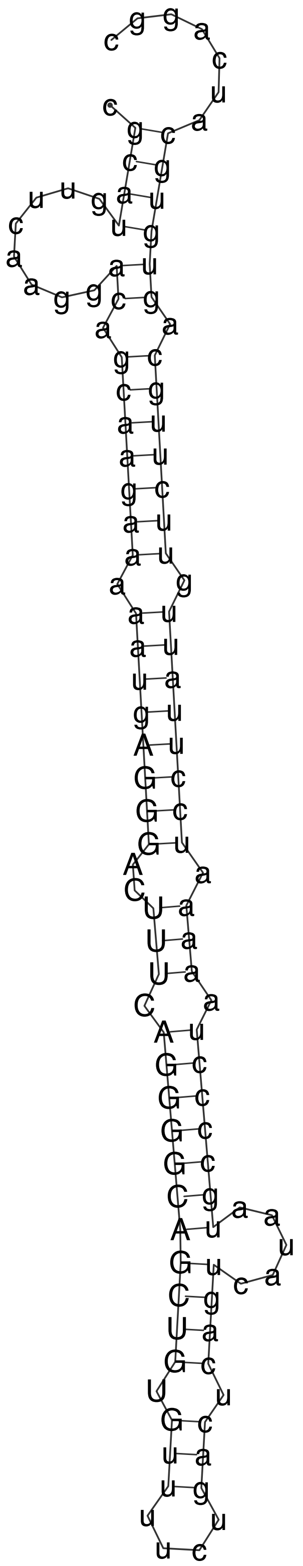

Genome:

APMK

Location:

fd0c4|gi|529608918|gb|
APMK01271231.1|:
2765-2889_Cricetulus_gris
eus_strain_17A/
GY_chromosome_7_chr7_cont
ig_1123_whole_genome_shot
gun_sequence

Mature sequence:

CACCACAGUGUGGUUUGGACGUGG

Precursor sequence:

ucagcggugccucccccacccucgc
ucuaaCACCACAGUGUGGUUUGGAC
GUGGccacagugccguacaaaccac
agugugcugcuggggcgggagcagg
acuuggaugguugagaggaagaca

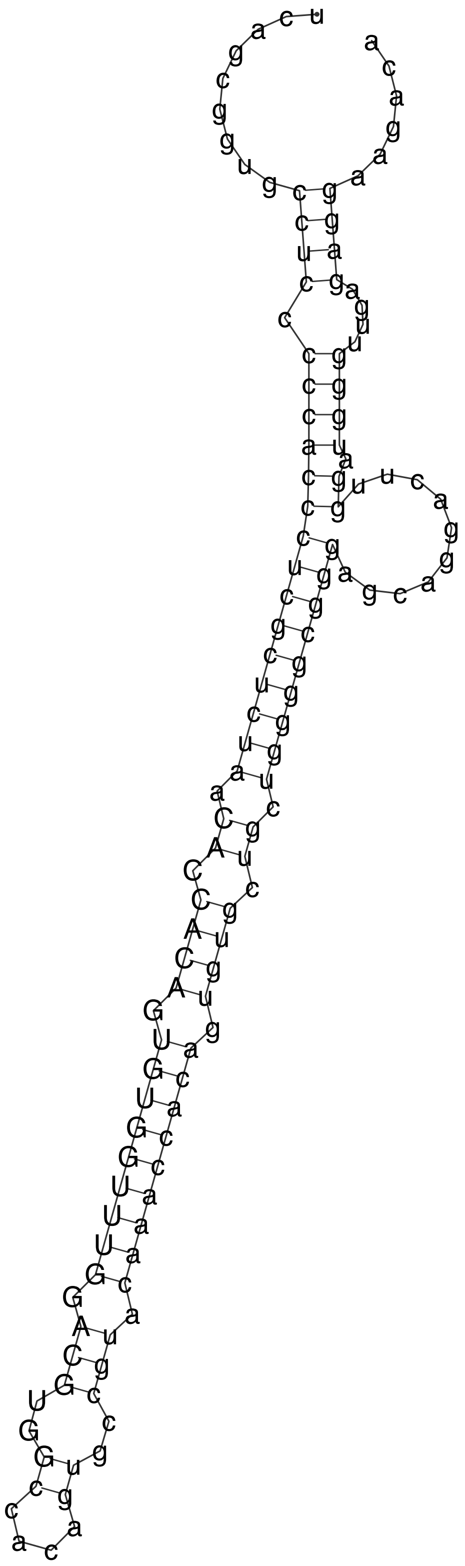

mmu-miR-598-5p

Genome:

APMK

Location:

e8410|gi|529899282|gb|APMK01130090.1|:c8661-8583_Cricetulus_griseus_strain_17A/GY_chromosome_1_chr1_contig_11798_whole_genome_shotgun_sequence

Mature sequence:

GCGGUGAUGCCGAUGGUGCGAGC

Precursor sequence:

gcugaugcugGCGGUGAUGCCGAUGGUGCGAGCugaaaaugggcugcuacgucaucgucgucaucguuaucaucaucau

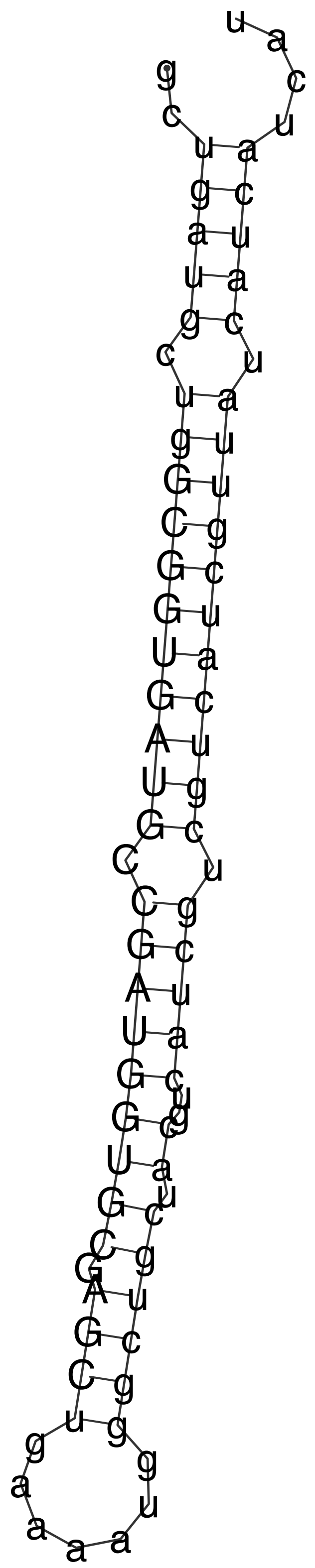

mmu-miR-674-5p

Genome:

APMK

Location:

de8e0|gi|529984327|gb|
APMK01089970.1|:c2494-239
5_Cricetulus_griseus_stra
in_17A/
GY_chromosome_6_chr6_cont
ig_128_whole_genome_shotg
un_sequence

Mature sequence:

GCACUGAGAUGGGAGUGGUGUA

Precursor sequence:

ggccuagucaucacccugagccuuG
CACUGAGAUGGGAGUGGUGUAaggc
uuaagugugcacagcucccaucuca
gaacaaggcucaggugugcacagcu

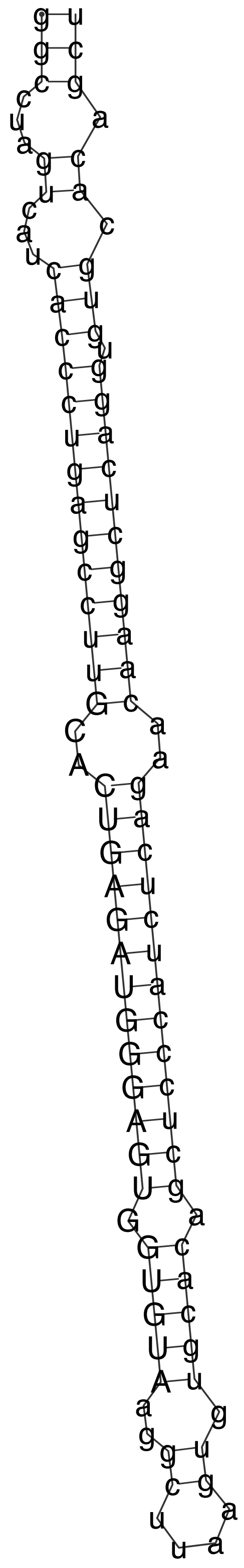

Genome:

APMK

Location:

b3910|gi|529508147|gb|APMK01314966.1|:2628-2746_Cricetulus_griseus_strain_17A/GY_chromosome_1_chr1_contig_16836_whole_genome_shotgun_sequence

Mature sequence:

CGGCUCUGGGUCUGUGGGGA

Precursor sequence:

cgggaggaugccucggugcggggcgcgucgccccucucaggccaccagagcccggagaccucagaaauuCGGCUCUGGGUCUGUGGGGAgcgaaaugcaacccaaagcccguuuccccc

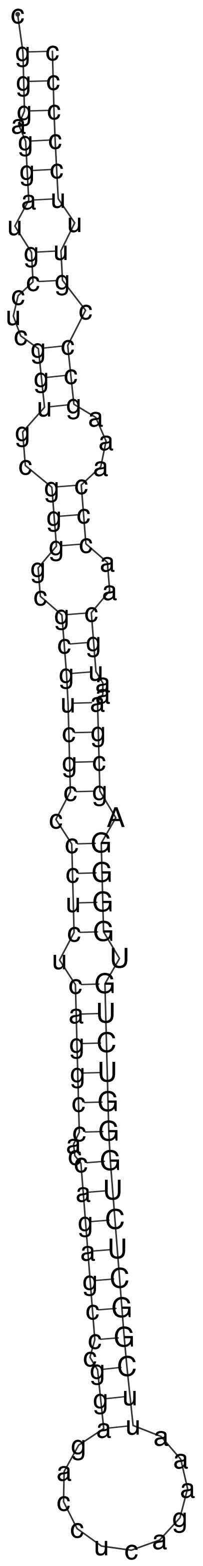

mmu-miR-99b-3p

Genome:

APMK

Location:

29563|gi|530137215|gb|APMK01015949.1|:1175-1244_Cricetulus_griseus_strain_17A/GY_unplaced_contig_5750_whole_genome_shotgun_sequence

Mature sequence:

CAAGCUCGUGUCUGUGGGUCCG

Precursor sequence:

ggcacccacccguagaaccgaccuugcggggccuucgccgcacaCAAGCUCGUGUCUGUGGGUCCGuguc

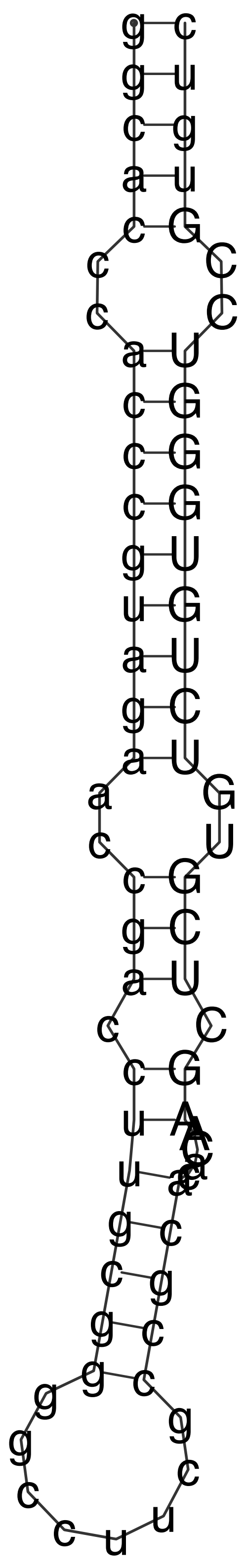

Genome:

APMK

Location:

f47a8|gi|530137215|gb|APMK01015949.1|:1175-1244_Cricetulus_griseus_strain_17A/GY_unplaced_contig_5750_whole_genome_shotgun_sequence

Mature sequence:

CACCCGUAGAACCGACCUUGCG

Precursor sequence:

ggcaccCACCCGUAGAACCGACCUUGCGgggccuucgccgcacacaagcucgugucuguggguccguguc

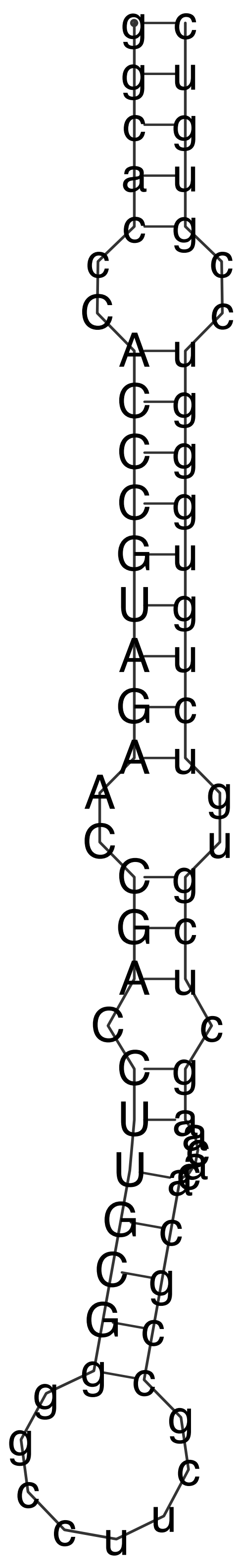

oan-miR-30d-5p

Genome:

K1-BB

Location:

83e9a|gi|342394869|gb|AFTD01238671.1|:443-546_Cricetulus_griseus_C40361668_1_whole_genome_shotgun_sequence

Mature sequence:

UGUAAACAUCCCCGACUGGAAGC

Precursor sequence:

guuagcuggcucucaggaagucugugucUGUAAACAUCCCCGACUGGAAGCuguaagccacuuccaagcuuucagucagauguuugcugccaccggcucuuccc

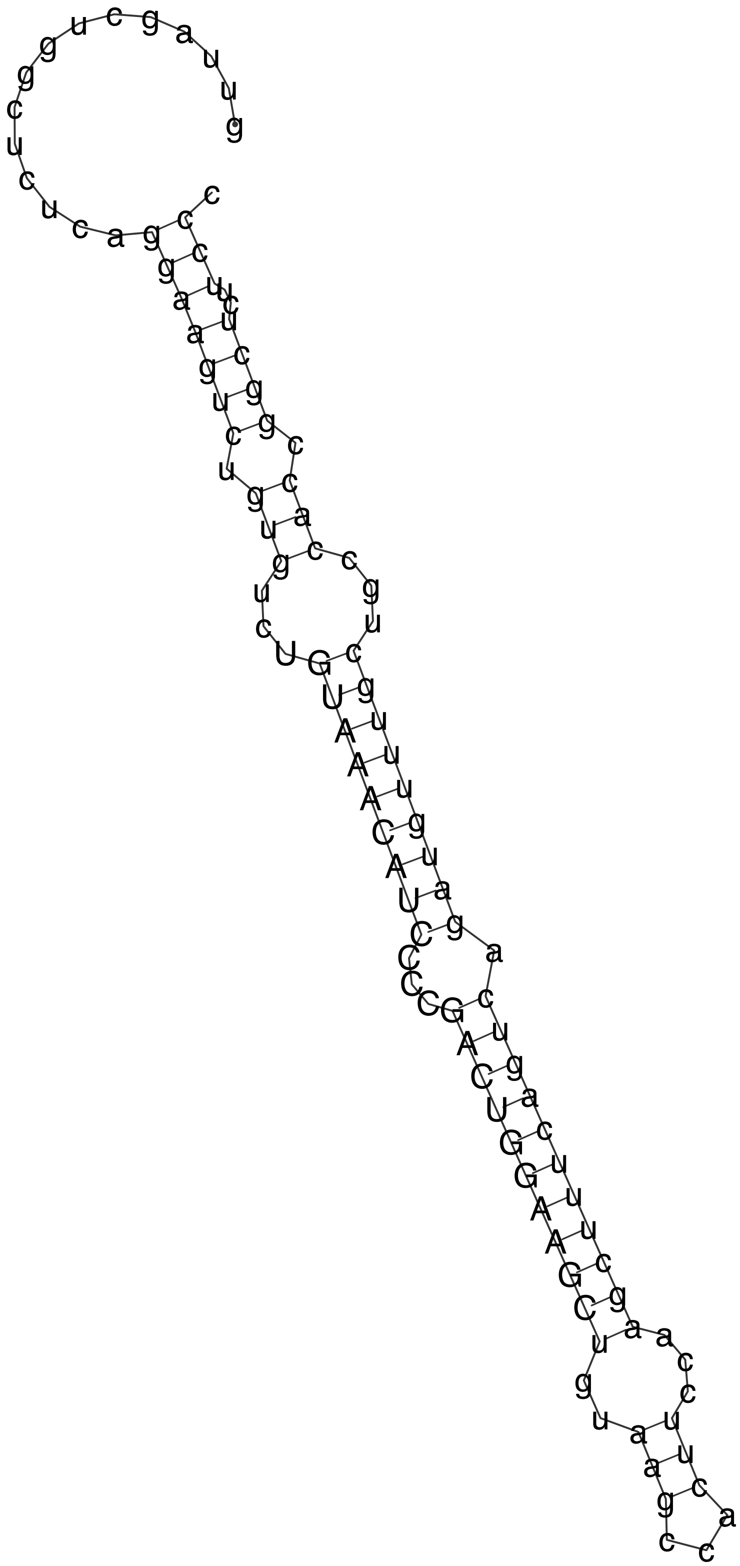

rno-miR-128-2-5p

Genome:

APMK

Location:

3959b|gi|530134202|gb|APMK01017492.1|:c342-259_Cricetulus_griseus_strain_17A/GY_chromosome_4_chr4_contig_37613_whole_genome_shotgun_sequence

Mature sequence:

GGGGGCCGAUGCACUGUAAGA

Precursor sequence:

ugugcagugggaagGGGGGCCGAUGCACUGUAAGAgagugaguagcaggucucacagugaaccggucucuuucccugcuguguc

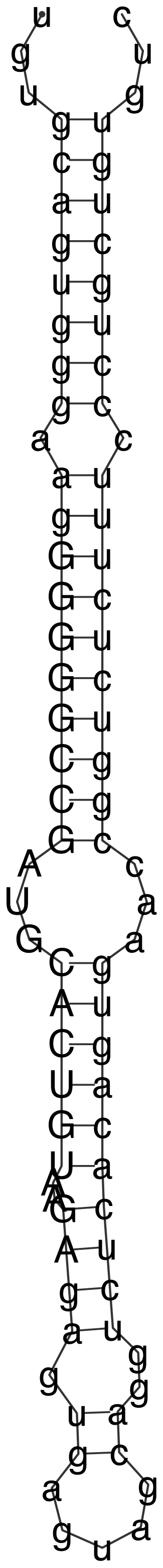

rno-miR-330-3p

Genome:

APMK

Location:

56e5b|gi|529980772|gb|
APMK01091321.1|:c149-53_C
ricetulus_griseus_strain_
17A/
GY_unplaced_contig_3040_w
hole_genome_shotgun_seque
nce

Mature sequence:

GCAAAGCACAGGGCCUGCAGAGA

Precursor sequence:

acccuuuggcgaucucugccucucu
gggccugugucuuaggcucuucagg
cucuaacgaGCAAAGCACAGGGCCU
GCAGAGAgguagcgcuccgcuc

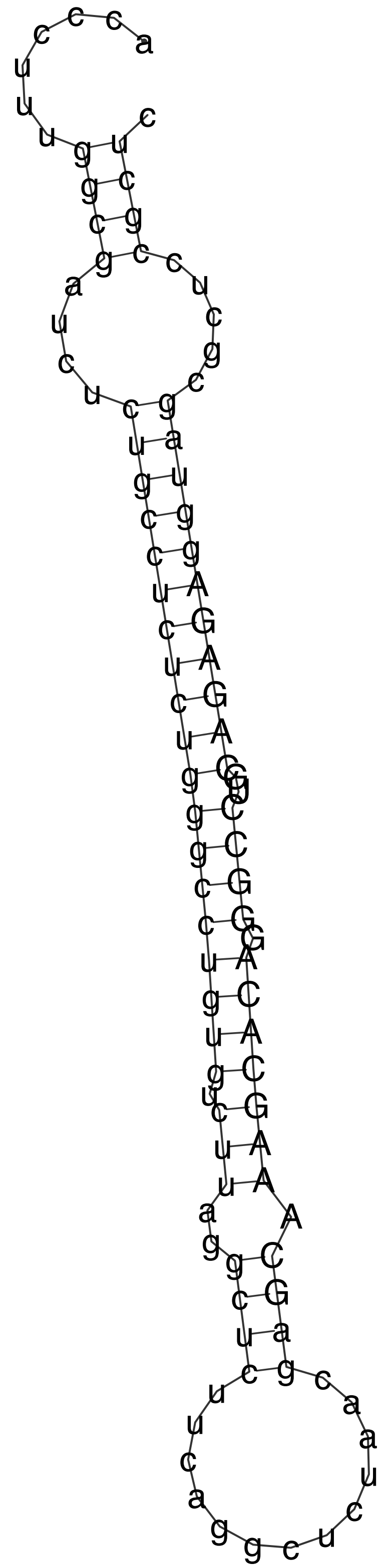

rno-miR-330-5p

Genome:

APMK

Location:

05cfd|gi|529980772|gb|
APMK01091321.1|:c149-53_C
ricetulus_griseus_strain_
17A/
GY_unplaced_contig_3040_w
hole_genome_shotgun_seque
nce

Mature sequence:

UCUCUGGGCCUGUGUCUUAGGC

Precursor sequence:

acccuuuggcgaucucugccUCUCU
GGGCCUGUGUCUUAGGCucuucagg
cucuaacgagcaaagcacagggccu
gcagagagguagcgcuccgcuc

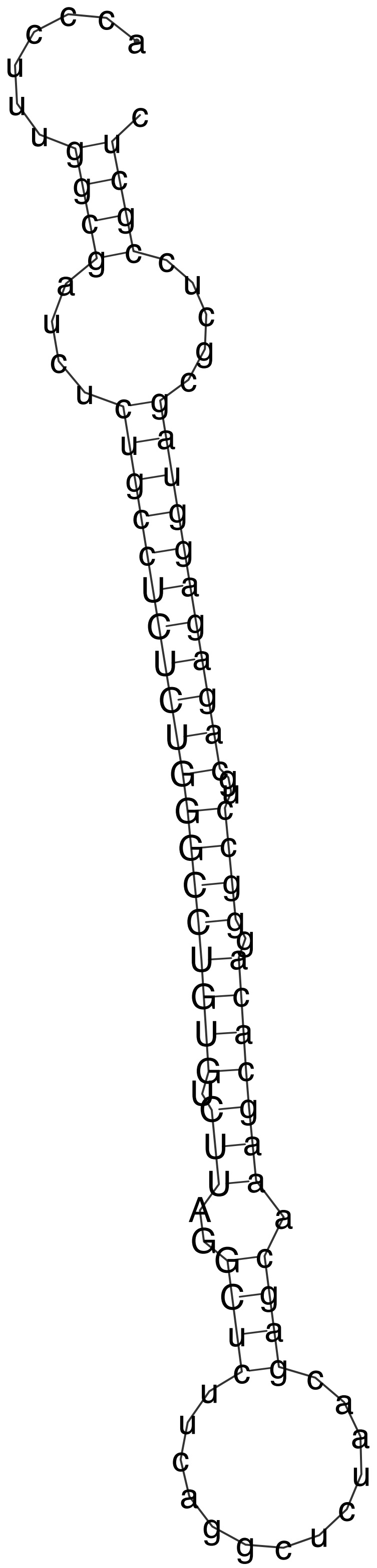

rno-miR-802-5p

Genome:

APMK

Location:

9a78a|gi|529611904|gb|
APMK01269960.1|:
19179-19274_Cricetulus_gr
iseus_strain_17A/
GY_chromosome_4_chr4_cont
ig_20845_whole_genome_sho
tgun_sequence

Mature sequence:

UCAGUAACAAAGAUUCAUCCU

Precursor sequence:

guucuauuauuugcaaUCAGUAACA
AAGAUUCAUCCUgugucaauugcac
aacacggagagucuuugucacucag
ugugauuaauagccuuccacu

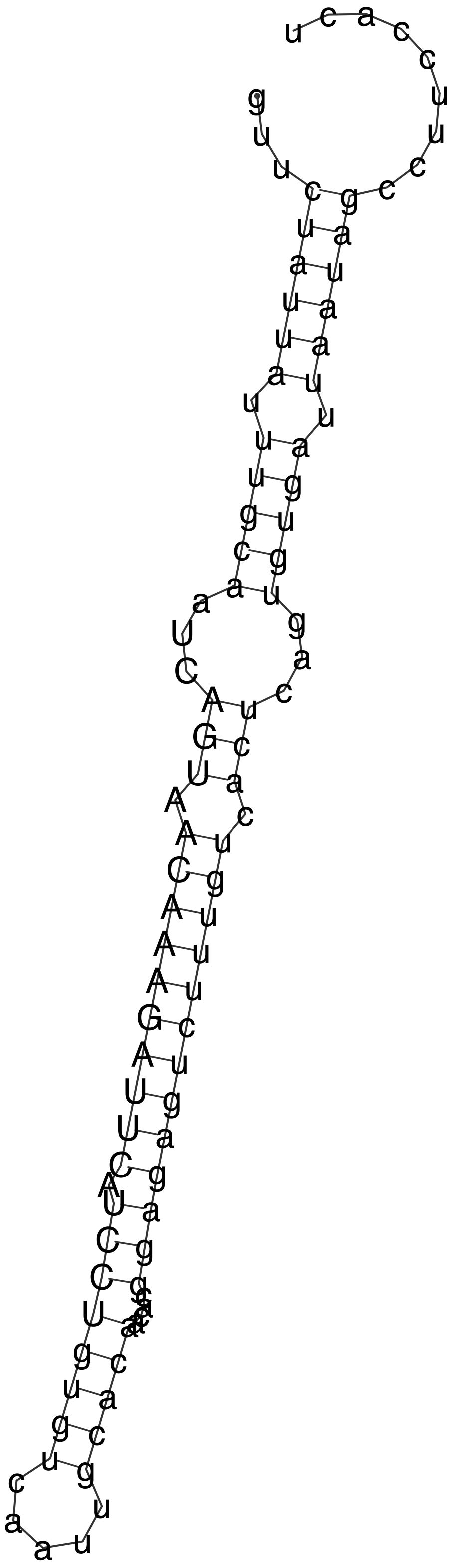

hsa-miR-18b-5p

Genome:

APMK

Location:

8974f|gi|529807703|gb|APMK01175604.1|:606-684_Cricetulus_griseus_strain_17A/GY_chromosome_X_chrX_contig_2805_whole_genome_shotgun_sequence

Mature sequence:

UAAGGUGCAUCUAGUGCAGUUAG

Precursor sequence:

ucuuguguUAAGGUGCAUCUAGUGCAGUUAGugaagcagcuuagacucuacugcccuaaaugccccuucucgcacaggc

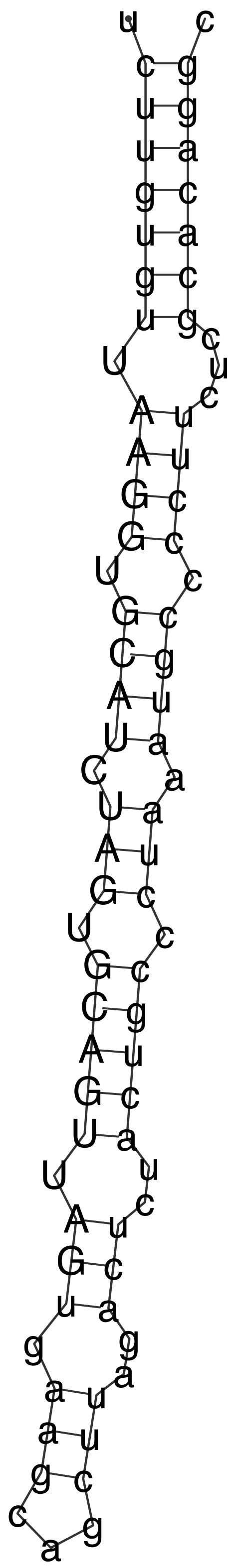

Genome:

APMK

Location:

dca45|gi|529807703|gb|
APMK01175604.1|:
829-902_Cricetulus_griseu
s_strain_17A/
GY_chromosome_X_chrX_cont
ig_2805_whole_genome_shot
gun_sequence

Mature sequence:

CAAAGUGCUCAUAGUGCAGGUAG

Precursor sequence:

guaguacCAAAGUGCUCAUAGUGCA
GGUAGuuuugcaucauucuacugca
cugugagcacuuccaguacucuug

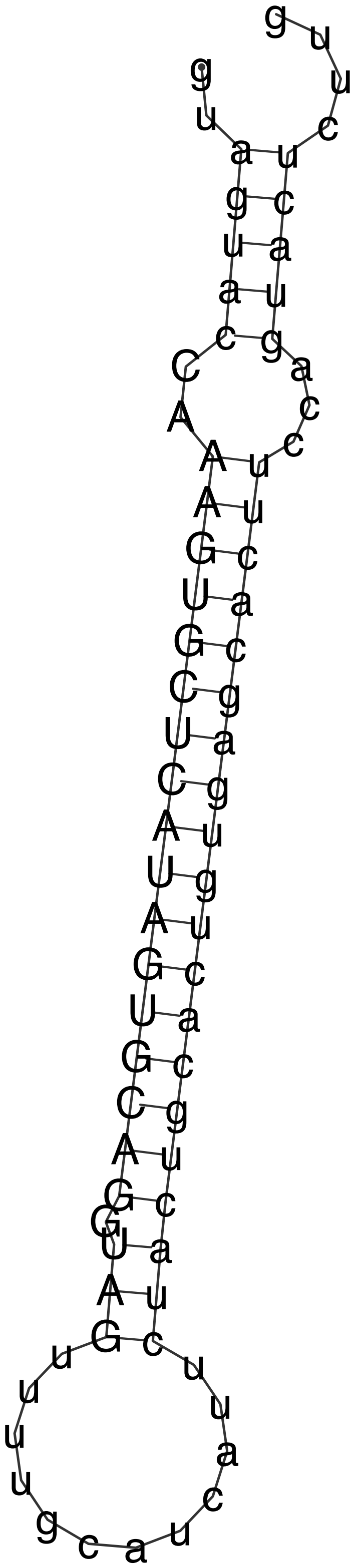

Supplement: Supplementary file 3 — Supplement 3. [file BIT-112-1488-s003.pdf]
